# Disposition of Proteins and Lipids in Synaptic Membrane Compartments Is Altered in Q175/Q7 Huntington’s Disease Mouse Striatum

**DOI:** 10.3389/fnsyn.2021.618391

**Published:** 2021-03-18

**Authors:** Maria Iuliano, Connor Seeley, Ellen Sapp, Erin L. Jones, Callie Martin, Xueyi Li, Marian DiFiglia, Kimberly B. Kegel-Gleason

**Affiliations:** Department of Neurology, Massachusetts General Hospital, Boston, MA, United States

**Keywords:** cholesteryl ester, lipidomics, PIP2, synapse, neurotransmitter receptors, electron microscopy, Huntingtin, density gradient centrifugation

## Abstract

Dysfunction at synapses is thought to be an early change contributing to cognitive, psychiatric and motor disturbances in Huntington’s disease (HD). In neurons, mutant Huntingtin collects in aggregates and distributes to the same sites as wild-type Huntingtin including on membranes and in synapses. In this study, we investigated the biochemical integrity of synapses in HD mouse striatum. We performed subcellular fractionation of striatal tissue from 2 and 6-month old knock-in Q175/Q7 HD and Q7/Q7 mice. Compared to striata of Q7/Q7 mice, proteins including GLUT3, Na^+^/K^+^ ATPase, NMDAR 2b, PSD95, and VGLUT1 had altered distribution in Q175/Q7 HD striata of 6-month old mice but not 2-month old mice. These proteins are found on plasma membranes and pre- and postsynaptic membranes supporting hypotheses that functional changes at synapses contribute to cognitive and behavioral symptoms of HD. Lipidomic analysis of mouse fractions indicated that compared to those of wild-type, fractions 1 and 2 of 6 months Q175/Q7 HD had altered levels of two species of PIP2, a phospholipid involved in synaptic signaling, increased levels of cholesterol ester and decreased cardiolipin species. At 2 months, increased levels of species of acylcarnitine, phosphatidic acid and sphingomyelin were measured. EM analysis showed that the contents of fractions 1 and 2 of Q7/Q7 and Q175/Q7 HD striata had a mix of isolated synaptic vesicles, vesicle filled axon terminals singly or in clusters, and ER and endosome-like membranes. However, those of Q175/Q7 striata contained significantly fewer and larger clumps of particles compared to those of Q7/Q7. Human HD postmortem putamen showed differences from control putamen in subcellular distribution of two proteins (Calnexin and GLUT3). Our biochemical, lipidomic and EM analysis show that the presence of the HD mutation conferred age dependent disruption of localization of synaptic proteins and lipids important for synaptic function. Our data demonstrate concrete biochemical changes suggesting altered integrity of synaptic compartments in HD mice that may mirror changes in HD patients and presage cognitive and psychiatric changes that occur in premanifest HD.

## Introduction

Patients with Huntington’s disease (HD) develop cognitive and motor dysfunctions accompanied by neuronal loss in the striatum and cortex. Altered synaptic connectivity of the basal ganglia underlies these deficits and is associated with remodeling dendritic morphology and spine loss of medium spiny striatal neurons where most synaptic contacts in the striatum occur (Graveland et al., 1985; Ferrante et al., 1991; Sotrel et al., 1991). Imaging studies in prodromal or early symptomatic HD patients and controls support a role for early changes in connectivity in cortex and in cortico-striatal networks in causing depression and trouble with executive processing (Wolf et al., 2008; Unschuld et al., 2012a, b). A profound loss of white matter also occurs early in HD (Tabrizi et al., 2011) and loss of white matter in premanifest HD carriers correlated with transcriptional changes in synaptic proteins (McColgan et al., 2018).

Studies in different HD mouse models also support the idea that synaptic dysfunction occurs early in disease significantly affecting the striatum (Nithianantharajah and Hannan, 2013). Decreased spine number and changes in dendritic morphology preceded neuron loss in human HD postmortem brain and in different HD mouse models (Graveland et al., 1985; Guidetti et al., 2001; Klapstein et al., 2001; Spires et al., 2004; Lerner et al., 2012). Using two-photon microscopy and a cranial window, Murmu et al. (2013) showed that spines have an increased rate of turnover and a lower survival rate in R6/2 HD mice, which overexpress a small fragment of mutant Huntingtin. Loss of excitatory terminals projecting from cortex to striatum occurs prior to striatal volume or neuronal loss in knock-in Q140 heterozygous mice (Deng et al., 2013). Shifts in spine types at 21 days and reduced spine density by 5 weeks occur in dorsal striatum of the knock-in Q175 (zQ175) compared to wild-type (McKinstry et al., 2014). These morphological changes are likely to contribute to altered synaptic function in HD. Differences in synaptic organization at the EM level have been reported in HD mice. Wheeler and colleagues found altered synaptic density and synaptic morphology in the striatum of 18-month old Q111/Q7 HD mice (Kovalenko et al., 2018). Based on studies in human postmortem brain and Q140 HD mice there are stages of synaptic loss with reduced inputs from cortex and thalamus to the striatum occurring early and output pathways of the striatum affected later (Reiner and Deng, 2018). In neurons, mutant Huntingtin collects in aggregates including at synapses (Li et al., 2003; Sapp et al., 2020), and distributes to the same sites as wild-type Huntingtin including on membranes and in synapses (Aronin et al., 1995). Wild-type Huntingtin has been shown to be associated with the presynaptic cytomatrix (Yao et al., 2014) and mutant Huntingtin can sequester Bassoon, a polyglutamine containing structural protein, and Piccolo away from the cytoskeletal matrix of the active zone (CAZ) in R6/1 mice and human HD brain (Huang et al., 2020).

Changes at plasma membranes and endosomes may contribute to dysfunction in HD. Studies in HD models showed that the HD mutation affects the organization of cholesterol enriched lipid rafts at plasma membranes where most growth factor signaling occurs in neurons and alters distribution of the Na^+^/K^+^ ATPase from detergent resistant membranes (Valencia et al., 2010). Displacement of the NMDAR 2b receptor subunit away from the synapse with no changes in protein levels was shown in YAC72 HD mice and correlated with altered current (Fan et al., 2007). Redistribution of AMPA receptors affect memory in HD models (Zhang et al., 2018). Glucose transport across the plasma membrane is impaired in primary HD neurons (McClory et al., 2014) and glucose utilization is both impaired in HD patients (Ciarmiello et al., 2006) and mirrors progression in premanifest HD (Tang et al., 2013). There is no evidence for loss of glucose transporter 3 (GLUT3) protein in HD cell and mouse models (McClory et al., 2014) or in HD brain (Gamberino and BrennanJr., 1994). However, mutant Huntingtin alters the endocytic recycling of Rab11 dependent cargo including transferrin receptor and GLUT3 back to the plasma membrane in primary Q140/Q140 HD neurons compared to wild-type without changing its protein levels (Li et al., 2009; McClory et al., 2014). Changes in GLUT3 subcellular localization have thus far not been demonstrated in animal models.

Cellular fractionation is a useful method to enrich for subcellular compartments including plasma membranes, organelles and synaptic profiles and for the low abundant proteins they may contain. With these preparations it is possible to determine by biochemical or enzymatic assays the effects of genetic mutations or drug treatments on altered trafficking within subcellular compartments [examples in HD (Fan et al., 2007)]. Moreover, the subcellular compartments where mutant proteins reside or have effects can be determined (Aronin and DiFiglia, 1992; De Rooij et al., 1996; Velier et al., 1998; Kim et al., 1999; Kegel et al., 2002; Fan et al., 2007). Sucrose has been commonly used to create density gradients to separate organelles including synaptosomes which are enriched in pre- and postsynaptic structures. In a study of synaptosomes isolated in a 0.35-2M discontinuous sucrose gradient from striatum of 6-month-old Q140/Q140 HD mice, mutant Huntingtin was detected and there were altered levels of pre- and post synaptic proteins compared to Q7/Q7 mice (Valencia et al., 2013). Gradients made using Percoll (colloidal silica), Nycodenz (a derivative of benzoic acid with three aliphatic hydrophilic side chains), and iodixanol (a water-soluble contrast agent) have been used for preparation of synaptosomes (Ford and Rickwood, 1982; Dunkley et al., 1986). These preparations are useful for retaining organelle osmolarity and viscosity throughout the density gradient (Zhang et al., 1998; Graham, 2015). OptiPrep which is a 60% solution of iodixanol has been used in cellular fractionation to isolate cell compartments for the study of disease models (Mamada et al., 2017) including HD (Velier et al., 1998; Kim et al., 1999).

In this study, we used OptiPrep as a medium for subcellular fractionation to examine the distribution of proteins and lipids in striatal lysates from Q7/Q7 and Q175/Q7 HD mice. Results showed that in comparison to those of Q7/Q7 mice, “synaptic” fractions 1 and 2 of 6-month old Q175/Q7 HD mice had differences from Q7/Q7 in the size and number of particles separated from these fractions and an altered distribution of membrane associated proteins, and the levels of PIP2, cholesterol ester, and other lipids involved in synapse signaling and maintenance. These changes may contribute to altered synaptic function in HD.

## Materials and Methods

### Animals

Knock-in heterozygous Q175/Q7 HD and Q7/Q7 C57BL/6J mice were obtained from The Jackson Laboratory (Stock 370832, JAX number 4156350). Mice were genotyped twice, once by PCR at Jackson Laboratory, as well as by western blot for Huntingtin protein. The animal protocol was approved by the MGH Subcommittee on Research Animal Care (SRAC)-OLAW #2004N000248. All procedures conform to the USD Animal Welfare Act, the “ILAR Guide for the Care and Use of Laboratory Animals,” and the PHS Policy on Humane Care and Use of Laboratory Animals. Animals were euthanized by CO_2_ followed by cervical dislocation and decapitation. Brains were extracted and rapidly dissected on ice to isolate the striata, which were immediately processed for density gradient ultracentrifugation.

### Human Brain

Unfixed, frozen human brain tissue was obtained from the Massachusetts General Hospital Neuropharmacology Laboratory Brain Bank and the Neuropathology Core of the Massachusetts Alzheimer Disease Research Center (P50 AG05134) and stored at −80°C. Post-mortem interval ranged from 4 to 48 h. Dorsal putamen was dissected from frozen blocks sectioned in the coronal plane.

### Striatal Sample Preparation and Cell Fractionation by Density Gradient Ultracentrifugation

The entire mouse striatum (both hemispheres) or a ∼2 cubic mm specimen of human putamen were homogenized with a Dounce homogenizer (10–20 strokes per sample) in 0.75 ml homogenization buffer (HB), containing 10 mM HEPES, 1 mM EDTA, 0.25M sucrose, and protease inhibitors (pH 7.4). Each sample was analyzed microscopically to verify that the nuclei had sheared from cellular components but had not ruptured. Additional 5 stroke increments were completed until an acceptable level of shearing had been obtained. Crude homogenate was spun for 10 min at 2,000g. The supernatant (S1) was collected and 0.45 ml was mixed with 50% OptiPrep (Sigma, cat #D1556) for a final concentration of 35% OptiPrep, then underlaid beneath the continuous density gradient. Continuous density gradients were prepared the day before by pouring discontinuous gradients (2.5 mL of 30%, 24%, 17%, and 10% OptiPrep diluted in HB buffer) in 14 × 89 mm Ultra-Clear centrifuge tubes (Beckman Coulter, 344059) and allowing them to diffuse and equilibrate overnight at 4°C. The loaded gradients were overlaid with HB to completely fill tubes and balance the rotor. Samples were centrifuged using a SW41 rotor in a Beckman L8-80 M Ultracentrifuge at 4°C for 2.5 h at 37,000 rpm with acceleration and deceleration set at 7. After centrifugation, 0.75 ml fractions were manually collected from top to bottom using a glass Pasteur pipette. Samples were then processed for SDS-PAGE and western blot analysis and stored at –80°C.

### SDS-PAGE and Western Blot

Fractions 1 through 15–16 were loaded by volume onto a 3–8% Tris-Acetate or 4–12% Bis-Tris gel using Tricine and MOPS running buffers, respectively, then transferred onto nitrocellulose via Trans-Blot Turbo transfer system (Bio-Rad). For some proteins, only *N* = 3 were investigated for all 1–16 fractions (including SNAP25). Fractions 1–3 were run on large format gels to compare samples from different animals on the same blot. *N* = 9 per group were examined in this manner for all reported probes. To prevent the aggregation of membrane proteins, samples were not boiled and gel electrophoresis chambers were run on ice. Blots were blocked in 5% milk in TBST (TBS + 0.1% Tween-20) then incubated overnight in blocking solution with diluted primary antibody. Blots were washed with TBST, incubated in blocking solution with diluted secondary antibody for 1 h, and then treated with SuperSignal West Pico Plus Chemiluminescent substrate (Pierce) for 5 min for band detection on film. We performed both film and digital readouts for many of the proteins detected. Examples of digital signals versus film signals are shown in Supplementary Figure 2A. For most proteins, film gave stronger, more consistent results compared to digital; the latter were in general agreement with film but for some proteins the digital signals were too low or absent to be useful. For film, we routinely used multiple exposure times (1, 10, or 30 s and 1, 3, and 30 min) and selected exposure times for quantification where the most intense signal among the fractions was not saturated. Signal for each fraction was standardized as a percentage of the total signal for all fractions and not based on absolute magnitude. For figures showing fractions 1–16, we used longer exposures so that the small amounts of protein in some fractions could be appreciated. Blots were stripped using ReBlot Strong Stripping Buffer (Millipore), and then blocked and re-probed for additional antibody detection. Antibody sources and dilutions can be found in the next section.

### Antibodies

Actin (Sigma, A4700) 1:400, Alpha Actinin 2 (Abcam, ab68167) 1:2000, AMPA Receptor 1 GluR1a (Alomone, AGC-004) 1:500, BIII Tubulin (Sigma, T8660) 1:2000, Calnexin (StressGen, SPA-860) 1:2000, DARPP32 (Abcam, 40801) 1:10,000, GABA(A) a1 Receptor (Alomone, AGA-001) 1:500, Glucose Transporter GLUT3 (Abcam, ab41525) 1:500, Na+/K+ ATPase (Affinity Bioreagents, MA3-915) 1:5,000, NMDA Receptor 2B GluN2B (Alomone, AGC-003) 1:500, PDE10a (Abcam, 177933) 1:2000, PSD95 (Cell Signaling, 2507S) 1:1000, SCN4B (Abcam, ab80539) 1:200, SNAP25 (BD Transduction Labs, 610366) 1:10,000, Transferrin (Thermo Fisher, 13-6800) 1:1000, VGlut1 (Synaptic Systems, 135302) 1:10,000, VGlut2 (Synaptic Systems, 135402) 1:10,000, XK (Aviva Systems Bio, ARP33809_P050) 1:1000, Huntingtin [Ab1, aa1-17, (DiFiglia et al., 1995)] 1:2000, poly-Q (1C2, EMD Millipore MAB1574) 1:1000. Horseradish peroxidase secondary antibodies (Jackson Immunoresearch) were diluted 1:5000.

### Pixel Intensity Quantification and Statistical Analysis

Signal intensity was measured using ImageJ software (NIH) and standardized to background signal. Signal from each fraction was standardized as a percentage of the sum of signal from all fractions. Wild-type and mutant Huntingtin were measured separately in the Q175/Q7 HD mice as detected with Ab1. In human post-mortem tissue, wild-type and mutant Huntingtin could not be distinguished using these assay conditions so the results are reported for total Huntingtin as detected with Ab1. Analyses of signal intensities were compared using a 2-tailed unpaired *t*-test. Fisher’s exact test was also used to compare peak signals in fractions 1 and 2. Statistical analyses were performed using GraphPad Prism version 7.00 for Windows (GraphPad Software, La Jolla, CA, United States). For Tukey-style box plots, the lower and upper hinges correspond to the first and third quartiles (the 25th and 75th percentiles). The upper whisker extends from the hinge to the largest value no further than 1.5 times the inter-quartile range (distance between the first and third quartiles) from the hinge. The lower whisker extends from the hinge to the smallest value no further than 1.5 times the inter-quartile range from the hinge. Data beyond the end of the whiskers are called “outlying” points and are plotted individually. The outlying values are included in the statistical analyses. A power analysis to determine group size for 95% confidence was performed using signal intensities for GLUT3 with the initial *n* = 3 per group data using G^∗^Power software which specified *n* = 8 per group. We fractionated *n* = 9 mice per group.

### Electron Microscopy

Aliquots from continuous density fractionation (fractions 1 and 2) of Q7/7 and Q175/Q7 mice (*N* = 9 per group) were fixed for 1 h in 4% PFA, then 4 μl was dropped onto Formvar grids and allowed to air dry before staining with 6% uranyl acetate and 6% lead citrate in closed chambers. Stained grids were examined at the Philly Dake Electron Microscope Facility using a JEOL JEM-1011 transmission electron microscope with AMTv601 software (Advanced Microscopy Techniques, Woburn, MA, United States) at 30,000×. For quantification, images were taken at 500×, using two grids per animal. Micrographs were cropped to standardize size in Photoshop to (1971 × 1900) by a blinded operator to select areas of micrograph devoid of shadows or artifacts. Images were imported into FIJI software (NIH), and thresholds adjusted using Renyi entropy method, then analyzed for particle number and size. Particle analysis was completed on all images using particle size greater than 1 pixel up to infinity pixels, all other settings were left as default. Results were exported to Excel and average particle size, median particle size and number of particles standardized to the number of fields were calculated in 7–45 fields per mouse. The number of images per group per fraction were, Q7/Q7 fraction 1: 36, 20, 8, 13, 7, 14, 11, 16, 16; Q175/Q7 fraction 1: 13, 16, 35, 39, 15, 32, 30, 27; Q7/Q7 fraction 2: 21, 15, 17, 38, 27, 14, 8, 18, 21; Q175/Q7 fraction 2: 13, 14, 34, 21, 7, 33, 45, 27,22. The mean particle size and number of particles was determined for each field and the results are reported as mean particle size per mouse (*N* = 9).

### Lipidomics

Samples were prepared and analyzed using ion switching mass spectrometry exactly as previously described (Breitkopf et al., 2017). Briefly, 100 μl of fractions 1 and 2 from each continuous iodixanol gradient were aliquoted into 10 ml glass scintillation vials. 750 μl methanol was added to each sample. Subsequently, 2.5 ml of Methyl tert-butyl ether (MTBE) was added to each sample, this mixture was incubated at room temperature on a platform shaker for 1 h. Following mixing, 625 μl of water was added to separate polar and non-polar phases. The upper phase was collected and placed into a HPLC vial with Teflon lids. The lower phase was re-extracted with 1 ml (MTBE/methanol/water, by volume 10/3/2.5). The upper MTBE phase of this re-extraction was collected and combined with initial upper phase. Pellets were dried and overlaid with N_2_ gas and stored at −20°C. Samples were hand delivered to Beth Israel Deaconess Medical Center mass spectroscopy core.

### Data Analysis of Lipids

For each fraction we calculated the ratio of the individual lipid intensity (area under the peak) to the intensity of total lipids per mouse and obtained the means of Q7/Q7 and Q175/Q7 HD mice (*N* = 9 per group) for each fraction and performed unpaired student’s *t*-tests assuming unequal variance. Peak intensity was translated into a percentage of sample total intensity, class total intensity and class total expressed as a percentage of the sample total. These values were compared between Q7Q/7 and Q175/Q7 samples using students *t*-test in Excel. We also compared the ratio of the intensities of all lipids in one class to the total lipids in fractions 1 and 2 and obtained mean values for Q7/Q7 and Q175/Q7 mice (*N* = 9 mice per group) for the sum intensity/total intensity for each class. *T*-tests were performed and significance was accepted at the *p* < 0.05 level. Additionally, *P*-value adjustments were made using Benjamini Hochberg correction in Rstudio.

### Amplex Red Quantification of Total Cholesterol in Sample

Small amounts of fractions 1 and 2 were analyzed using the Amplex red cholesterol kit (Invitrogen, 3–5 μl for 6-month samples and 10 μl for 2-month samples). The Tukey-style box plot shows the median and interquartile ranges for both Q175/Q7 and Q7/Q7 in fractions 1 and 2.

### Annotation of Lipids

Phospholipids were annotated as Lipid Class (total fatty acid chain length: total number of unsaturated bonds). If acyl chains could be identified, carbon length of each fatty acyl chain and number of unsaturated bonds is listed in tables; however, assignment of the sn-1 and sn-2 positions is ambiguous (ex., PA 38:4 (18:0/20:4). Glycerophosphate or phosphatidic acid (PA), glycerophosphatidylcholine (PC), glycerophosphatidylethanolamine (PE), glycerophosphatidylglycerol (PG), glycerophosphatidylinositol (PI), glycerol-phosphatidylmethanol (PMe), glycerophosphatidylserine (PS), sphingomyelin (SM), and cardiolipin (CL). Lyso-PA (LPA), lyso-PC (LPC), lyso-PE (LPE), lyso-PG (LPG), lyso-PI (LPI), ceramide (Cer), sphingosine (So), sphingosine phosphate (SoP), methyl-PC (MePC), PI bis phosphate (PIP2). Glycerolipids: diacylglycerol, (DG). monoacylglycerol (MG), and triacylglycerol (TG). Neutral lipids: Fatty acids (FA), acylcarnitine (Acca), sterol (ST), cholesteryl ester, (ChE), zymosteryl (ZyE), wax ester (WE), Coenzyme (Co). E, ether linkage; P, plasmalogen; Ox, oxidized lipid; P, phosphate; Ganglioside (GM, GD).

## Results

### Analysis of OptiPrep Fractions of Q7/Q7 and Q175/Q7 HD Striatum for Presence of Standard Subcellular Markers and Huntingtin

**FIGURE 1:**
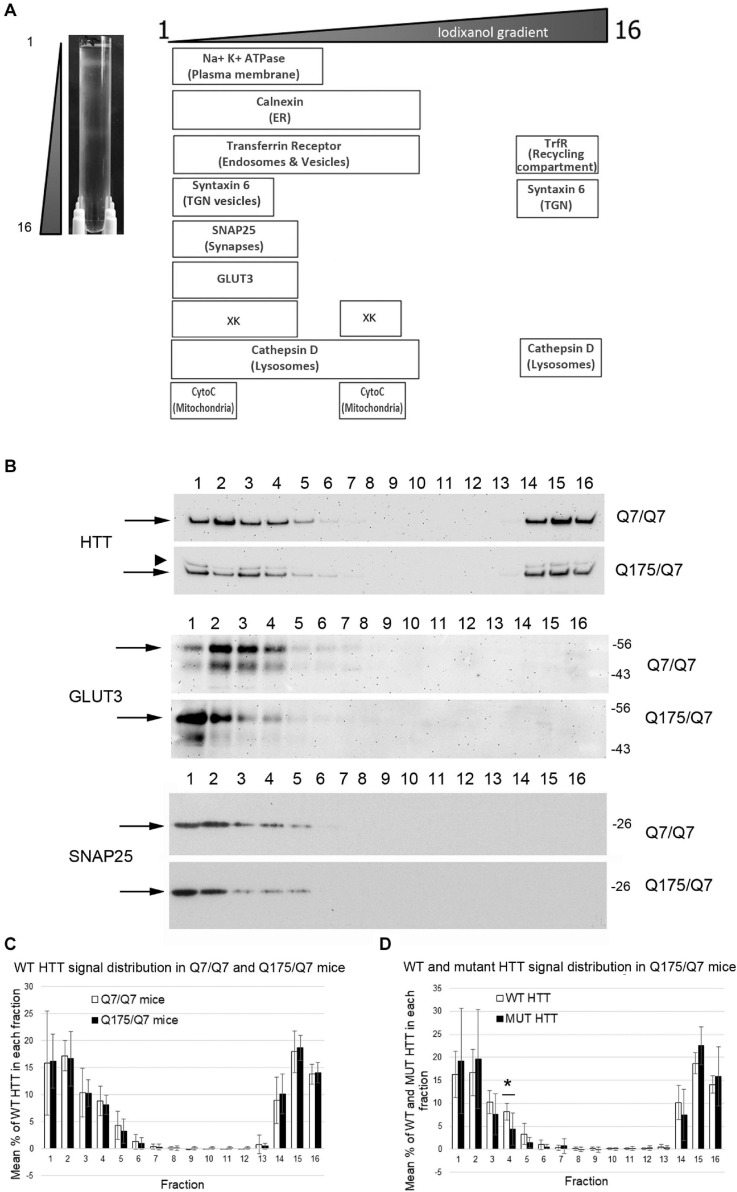
Subcellular fractionation by continuous iodixanol density gradient ultracentrifugation of 6-month old Q7/Q7 and Q175/Q7 HD mouse striatum. **(A)** Striata were fractionated as described in section “Materials and Methods.” Picture at left shows ultracentrifugation tube after equilibration of compartments by centrifugation; banding pattern indicates successful fractionation of cellular components. Fractions 1–16 were removed from top to bottom as indicated. Fractions were assessed by SDS-PAGE and western blot analysis using the cell compartment markers indicated at top right to ensure complete separation. Equal volumes of each fraction were loaded per lane. **(B)** Representative images of western blots detecting Huntingtin (HTT) (WT and mutant (arrowhead)), GLUT3, and SNAP25. HTT distributes to light fractions 1–5 and co-fractionates with a marker of pre-synaptic compartment, SNAP25 and to dense fractions 14–16. GLUT3 co-fractionates in fractions 1–4 and shows a marked shift in the peak signal intensity between Q7/Q7 and Q175/Q7 HD resulting in an increase in signal for GLUT3 in Q175/Q7 in fraction 1 and a reduction in fractions 2–4. **(C)** Pixel intensity analysis of western blots for WT HTT. Bar graph shows mean percent ± SD of WT HTT in each of 16 fractions as a percent of total WT HTT signal summed across all fractions (1–16). White bars show WT HTT in Q7/Q7 mice and black bars show WT HTT in Q175/Q7 striata. There was no significant difference in WT HTT signal as percent of total signal between Q7/Q7 and Q175/Q7 mice for any fraction. **(D)** Pixel intensity analysis of western blots for mutant HTT and WT HTT in fractions from Q175/Q7 striata. Bar graph shows percent ± SD of WT HTT (white bars) and mutant HTT (black bars) signal in each of 16 fractions as a percent of total WT and mutant HTT signal summed across all fractions (1–16) in Q175/Q7 6-month old mice. There is a significant difference between WT and mutant HTT signal as a percent of total signal in fraction 4 (*p = 0.0266, unpaired *t*-test, *N* = 7).

We sought to determine if the HD mutation altered the subcellular distribution of Huntingtin and other proteins in the striata of HD Q175/Q7 mice. Continuous iodixanol gradients were used to fractionate the post nuclear supernatants of Q7/Q7 and Q175/Q7 HD striata (*N* = 3 mice per genotype) These supernatants contain numerous membrane-bound compartments such as synaptosomes, light endosomes, endoplasmic reticulum (ER), mitochondria, peroxisomes, plasma membrane (PM) and recycling compartments, as well as cytosol (Figure 1A and Supplementary Figure 1). After ultracentrifugation, 16 fractions from each gradient were collected and analyzed by SDS-PAGE and western blot to establish the distribution of proteins known to be found in different compartments including PM (Na^+^/K^+^ ATPase), ER (Calnexin), recycling compartment (Transferrin receptor), and mitochondria (cytochrome C). The boxes in the diagram in Figure 1A summarize the distribution of proteins in fractionations from Q7/Q7 striata. Na^+^/K^+^ ATPase distributed to the least dense fractions (#1–6). Transferrin receptor distributed to fractions 1–10, but also to dense fractions (14–16) as expected for a protein that is found both on the plasma membrane and in the perinuclear recycling compartment which distributes to the denser fractions. In contrast, cytochrome C distributed to fractions 1 and 2 peaking in fraction 2 and to the center of the gradient (middle fractions 7–9) showing good separation of organelles on the gradients. Signal for Calnexin was also found in the lighter fractions but had a wider distribution (fractions 1–10) than the PM marker Na^+^/K^+^ ATPase. The protein XK, which has been implicated in non-Huntington’s chorea (Park and Neiman, 2020), distributes to light fractions 1–5 and to the center of the gradient (fractions 7–9). Syntaxin 6, a marker of trans-golgi network (TGN) and TGN-derived light vesicles distributes to fractions 1–4 and dense fractions 14–16 in Q7/Q7 mice. The lysosomal protease Cathepsin D fractionated to light fractions 1–9 with lower levels in fractions 3 and 4 and to heavy fractions 14–16. Wild-type Huntingtin signal occurred in fractions 1–6 and 14–16 with a distribution profile highly similar to Transferrin receptor, as previously described for human fibroblasts (Velier et al., 1998; Figures 1B,C). The location of synaptic components was identified by probing for the presynaptic marker SNAP25 which showed strong signal distributed to fractions 1 and 2 with lower levels in fractions 3–5 for Q7/Q7 striatum. Fractionations from Q175/Q7 HD striatum showed similar profiles for most proteins with no significant differences or trends in distribution for Calnexin, Transferrin receptor, and cytochrome C. The presynaptic marker SNAP25 appeared slightly shifted from its normal peak distribution in fraction 2 in Q7/Q7 to fraction 1 for Q175/Q7 in 2 of *N* = 3 mice (Figure 1B and Supplementary Figure 1).

In fractions from heterozygous Q175/Q7 striatum, wild-type Huntingtin distributed nearly identically to wild-type Huntingtin from Q7/Q7 striatum (Figures 1B,C). Mutant Huntingtin from Q175/Q7 striatum also distributed with a very similar profile, although a significant reduction of mutant Huntingtin occurred in fraction 4 (Figure 1D, ^∗^*p* = 0.027, unpaired *t*-test, *N* = 7). The distribution of Na^+^/K^+^ ATPase appeared to be changed in Q175/Q7 fractions compared to Q7/Q7 (Supplementary Figure 1). We also looked at the distribution of the neuronal specific protein GLUT3, presynaptic proteins such as VGLUT1 and VGLUT2, and post synaptic proteins. For PSD95 and VGLUT2 we have reported significant reductions in levels in synaptosomes of Q175/Q7 versus Q7/Q7 striatum; no change in protein levels was observed for Na^+^/K^+^ ATPase, SNAP25, and VGLUT1 (Sapp et al., 2020). We observed that several membrane-associated proteins that distribute to fractions 1–4 appeared altered from their normal distribution of peaking in fraction 2 in Q7/Q7 mice to peaking in fraction 1 in Q175/Q7 HD mice, (Figure 1B, shown for GLUT3 and Supplementary Figure 1B) similar to SNAP25. No changes were observed in fractions 3 and 4 between wild-type and Q175/Q7 mice. We chose to quantify changes in the peak signals, therefore one caveat to our results is that we may have missed changes that might occur in fractions where low levels of proteins distributed as they may be out of the linear range that we measured.

### The Presence of the HD Mutation Alters Distribution of Membrane Associated Proteins in Synaptic Compartments of Q175/Q7 HD Mice

**FIGURE 2:**
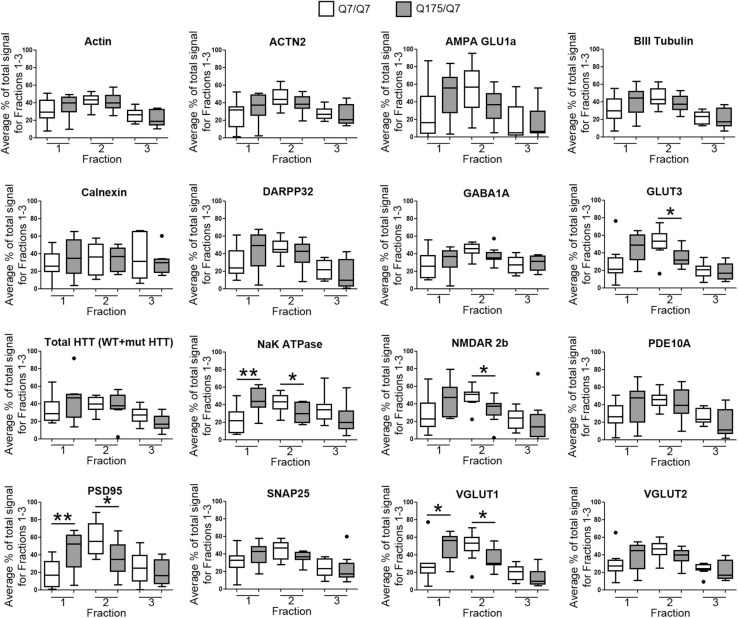
GLUT3, VGLUT1, Na^+^/K^+^ ATPase, NMDAR 2b, and PSD95 from 6-month old Q175/Q7 striata show altered distribution after fractionation compared to that of Q7/Q7 mouse striata. Equal volumes of fractions 1–3 from Figure 1 were run on large format SDS-PAGE gels and analyzed by western blot and ECL for proteins indicated. We performed both film and digital readouts for the many of proteins detected. We selected exposure times for quantification where the most intense signal among the fractions was not saturated. Signal for each fraction was standardized as a percentage of the total signal for all fractions and not based on absolute magnitude. Pixel intensity quantification was performed using ImageJ software (NIH) and graphed as percent of total signal summed for each protein in fractions 1–3 shown as a percentage of 100% Q7 or a percentage of Q175 100% (*N* = 9 mice per group, *p < 0.05, **p < 0.01, unpaired *t*-test). Graphs show Tukey-style box plots where the lower and upper hinges correspond to the first and third quartiles (the 25th and 75th percentiles) as described in section “Materials and Methods.” Data beyond the end of the whiskers are called “outlying” points and are plotted individually. The outlying values are included in the statistical analyses. Proteins with altered subcellular distribution include GLUT3, Na^+^/K^+^ ATPase, NMDAR 2b, PSD95 and VGLUT1. See Supplementary Figure 2B for representative western blot images.

Results established that in HD mice there was a minor difference in the subcellular distribution of wild-type and mutant Huntingtin, but the presence of the HD mutation changed the subcellular distribution of some other proteins in the lighter fractions 1–2. To quantify the change in distribution of proteins in the light fractions, we ran fractions 1–3 from each mouse on large format gels (26 lanes) and increased the sample size to *N* = 9 mice based on a power analysis (G^∗^Power). Figure 2 shows graphical representations of pixel intensity quantification results from western blots for 16 proteins; representative images of western blots are shown in Supplementary Figures 2A,B. A shift in the peak for several proteins did occur. A significant reduction of signal was observed in fraction 2 in Q175/Q7 mice for GLUT3, Na^+^/K^+^ ATPase, NMDAR 2b, PSD95, and VGLUT1 (Table 1) compared to Q7/Q7 mice, with a contingent significant or near significant increase in signal in fraction 1. Although our previous results with *n* = 3 mice showed that SNAP25 had a trend to shift from fraction 2 in Q7/Q7 to fraction 1 in Q175/Q7 HD (see also Figure 1B), the change was not significant with a larger sample size (Figure 2; *p* = 0.0527, unpaired *t*-test, *N* = 9). We also analyzed distribution of proteins well-known to have reduced levels in HD mouse models including DARPP32, PDE10A, and SCN4B (Sapp et al., 2020). Although the levels of these proteins were all reduced in total signal on the western blots of fractions from Q175 compared to wild-type, their peak distribution was in lower fractions (3–7) and no shift in their percent distribution in the gradient occurred. We also looked at XK, a protein that when mutated produces chorea (Jung et al., 2003; Urata et al., 2019) and found no change in levels or distribution occurred in fractionations for XK for Q175/Q7 HD compared to Q7/Q7 striatum.

**TABLE 1:** Statistical analysis for protein detection in fractions 1–3.

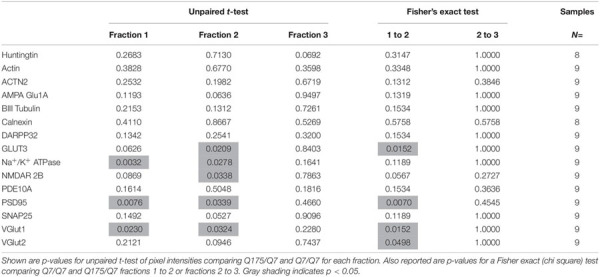		

To determine if changes in the distribution of membrane associated proteins occurred earlier than 6 months in the Q175/Q7 HD mice, we prepared fractions from striatum of 2-month old mice. There was a similar distribution of Huntingtin and the cellular markers in Q175/Q7 HD compared to Q7/Q7 mice (Supplementary Figure 3B). There were also no shifts observed between fractions 1–4 in the proteins altered at 6 months (Supplementary Figure 3A). These results suggest that the alterations in membrane associated proteins in the striatal synaptic fractions seen at 6 months are age dependent and not due to altered development or an early degenerative event.

These results show an age dependent change in the intracellular distribution of select proteins in Q175/Q7 HD mouse striatum that occurs independent of protein levels. These changes might imply a change in the physical nature of the compartment/organelle or a change in trafficking of proteins from one organelle to another.

### Mass Spectrometry of Lipids in Fractions 1 and 2 Reveals Marked Changes in Levels of Numerous Lipids Including Acyl Carnitine, Cardiolipin, Cholesterol Ester, Phosphatidic Acid, and PIP2 in Q175/Q7 HD Mice

**FIGURE 3:**
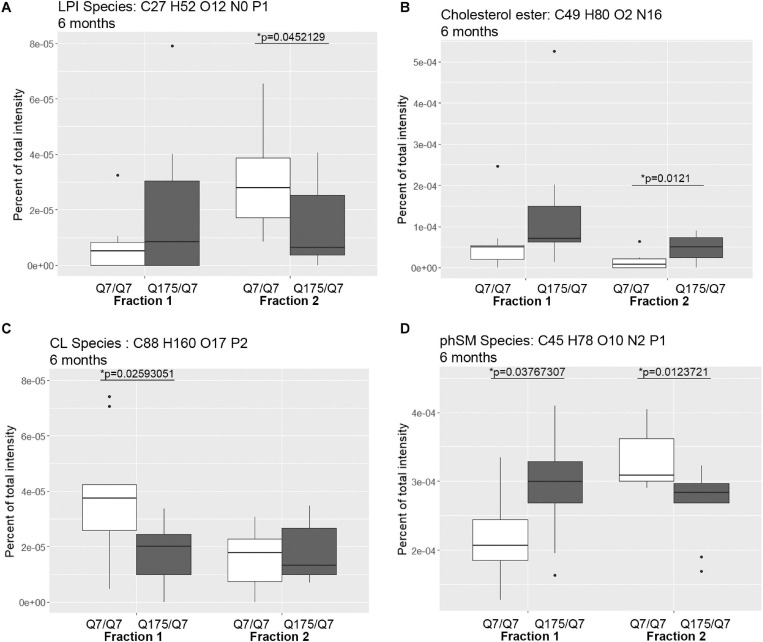
Levels of selected individual species of lipids detected by LC-MS/MS in fractions 1 and 2 of continuous density gradients of fractionated 6 months old Q175/Q7 and Q7/Q7 striata. Graphs show Tukey-style box plots where the lower and upper hinges correspond to the first and third quartiles (the 25th and 75th percentiles) as described in section “Materials and Methods.” Data beyond the end of the whiskers are called “outlying” points and are plotted individually. Outliers were not removed from statistical analysis. NOT DETECTED indicates species was not detected using the modified 80% rule (Yang et al., 2015). Changes at 6 months are shown for specific species of **(A)** lyso-phosphoinositol (LPI), **(B)** Cholesterol ester, **(C)** Cardiolipin (CL), and **(D)** Sphingomyelin, phytosphingosine (phSM). All significant changes are shown in Supplementary Tables 1–8. Comparisons were made between genotype for each fraction (^∗^*p* < 0.05, unpaired *t*-test, *N* = 9 per group).

In view of the changes described above in subcellular distribution of some membrane associated proteins in fractions 1 and 2 of the 6 months old HD mice, we addressed by mass spectrometry if lipid composition of these fractions was altered by the HD mutation. Lipidomic analysis of fractions 1 and 2 from 6 months old mice showed a consistent high yield of lipid species in all the samples examined. The number of individual lipids species identified in each Q175/Q7 HD mouse ranged from 544 to 816 in Q175/Q7 HD mice and from 613 to 815 in Q7/Q7 mice. The number of subclasses of lipids in the fractions from the Q175/Q7 HD mice varied from 26 to 34 and in the Q7/Q7 mice from 30 to 34. Bar graphs in Supplementary Figure 2 show different subclasses of lipids grouped by their relative abundance and compared between Q7/Q7 and Q175/Q7 HD mice at 6 months (Supplementary Figure 4A, Glycerophospholipids and Supplementary Figure 4B, “Other Lipids”; *N* = 9 per genotype). The abundance of the major Glycerophospholipid subclasses which compose cell membranes did not differ by genotype in either fraction (PC, PE, SM, PS, PI, PA, PG; Supplementary Figure 4A). Only three changes were identified among all lipid subclasses detected (Table 2). In fraction 1, phosphatidylethanol (PEt) was significantly lower in Q175/Q7 mice versus Q7/Q7 (*p* = 0.0469, unpaired *t*-test, *N* = 9; Table 2 and Supplementary Figure 4A, middle panel). In fraction 2, LPI was significantly decreased in Q175/Q7 mice compared to Q7/Q7 (*p* = 0.0452, unpaired *t*-test, *N* = 9; Table 2 and Supplementary 4A, bottom panel); this change was due to only one species detected and although not significantly different in fraction 1, an inverse change occurred in fraction 1 with more LPI in Q175/Q7 than in Q7/Q7 (Figure 3A), showing a similar redistribution pattern to the proteins in Figure 2. Intriguingly, a significant increase (nearly 3.5 fold) in Sterol (ST) occurred in Q175/Q7 compared to Q7/Q7 in fraction 2 (Table 2 and Supplementary Figure 4B). The only species measured in this subclass was cholesterol ester (ChE, also known as cholesteryl ester) (Figure 3B). In contrast to LPI, cholesterol ester increased in fraction 2 and trended to increase in fraction 1 in Q175/Q7 samples, suggesting an overall increase of cholesterol ester in the HD fractions instead of a shift from one fraction to another. To further validate the change in cholesterol ester levels we measured total cholesterol in fractions 1 and 2 using an Amplex colormetric assay which measures both cholesterol and cholesterol ester. Results showed a trend toward the same results as that found by LC MS (increased in both fractions 1 and 2 for Q175/Q7 versus Q7/Q7), but did not reach significance (Supplementary Figure 5).

**TABLE 2:** Altered lipid classes.

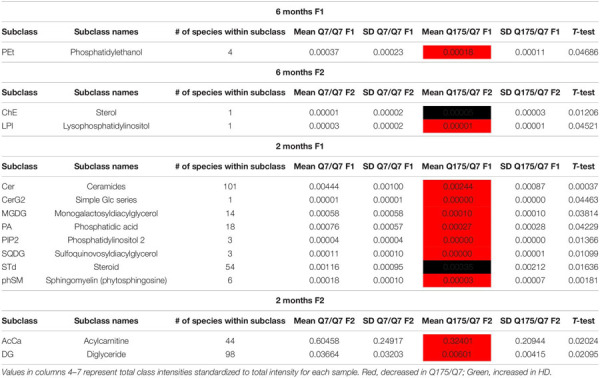		

Looking at individual lipid species at 6 months revealed numerous changes (Supplementary Tables 1, 2) in addition to LPI and cholesterol ester. A species of cardiolipin (CL), which is a component of mitochondria, was significantly decreased Q175/Q7 in fraction 1 compared to Q7/Q7 (Figure 3C). In fact, the cardiolipin subclass which includes numerous species also trended to be lower in fraction 1 but did not reach significance (Supplementary Figure 4A, bottom panel). Since no changes in fraction 2 were measured, this result suggests either a loss of this species of cardiolipin from mitochondria or possibly a loss of mitochondria themselves from fraction 1, possibly from the HD synapses. Another interesting change was for a species of phytosphingosine (phSM; Figure 3D and Supplementary Tables 1, 2) which changed inversely in fractions 1 and 2 with an increase in fraction 1 and a decrease in fraction 2, similar to LPI.

**FIGURE 4:**
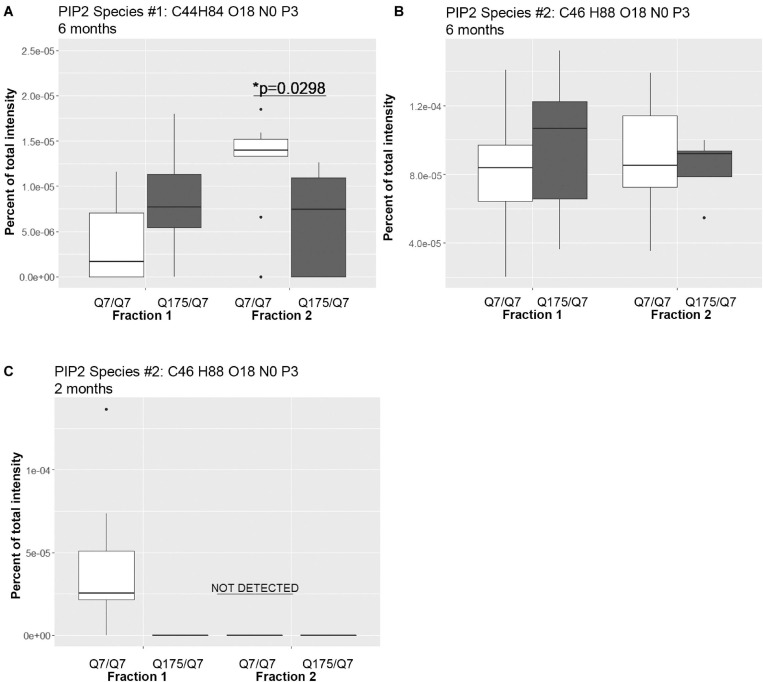
Levels of PIP2 species detected by LC-MS/MS in fractions 1 and 2 of continuous density gradients of fractionated Q175/Q7 and Q7/Q7 striata. Graphs show Tukey-style box plots where the lower and upper hinges correspond to the first and third quartiles (the 25th and 75th percentiles) as described in section “Materials and Methods.” Data beyond the end of the whiskers are called “outlying” points and are plotted individually. Outliers were not removed from statistical analysis. NOT DETECTED, indicates species was not detected using the modified 80% rule (Yang et al., 2015). **(A,B)** Levels of the two PIP2 species detected at 6 months in fractions 1 and 2. In **(A)**, there was a significant increase in PIP2 species #1 (C44 H84 O18 N0 P3) in fraction 2 between Q7/Q7 and Q175/Q7 (^∗^*p* < 0.05, unpaired *t*-test, *N* = 9 per group), whereas there was no significant difference in the second species in either fraction between genotypes **(B)**. **(C)** Levels of PIP2 species #2 (C46 H88 O18 N0 P3) detected in fractions 1 and 2 at 2 months. This was the sole species detected at 2 months and was only detected in fraction 1 from Q7/Q7 striata.

A significant change in levels of one species of PIP2 occurred (Figure 4 and Supplementary Table 2). PIPs are of interest because they are involved in important signal transduction pathways and wild-type and mutant Huntingtin bind them (Kegel et al., 2005, 2009). They can act as secondary messengers (as in PLC signaling) or to recruit protein complexes to membranes (as in PIK3 signaling). Using the MBTE extraction method, three species of the signaling glycerophospholipids PIP2 were detected (Figure 4). One PIP species (C44 H84 O18 N0 P3) was significantly lower in Q175/Q7 fraction 2 compared to Q7/Q7 (Figure 4A) and was much less abundant than a second species that was not significantly different (C46 H88 O18 N0 P3) (Figure 4B). Because the mass of the phospholipid head group is identical, using this method it is not possible to identify the exact PIP2 without further study using standards and methods designed to optimize PIP extraction and analysis (HPLC or GC methods). However, given the relative abundance of the second PIP2 species (C46 H88 O18 N0 P3) compared to the first (note *y*-axis in Figure 4B compared to A), we speculate that (C46 H88 O18 N0 P3) is PI (4,5)P2 which is known to be a fold more abundant than PI (3,4)P2 and PI(3,5)P2. Changes in individual species of PE, PC and PS reflected subtle changes in acyl chain usage rather than shifts between compartments at 6 months.

Next, we performed a lipidomic analysis in fractions from 2 months old mice to see if changes observed in HD mice at 6 months occur early or were degenerative or compensatory. Bar graphs in Supplementary Figure 6 show different subclasses of lipids grouped by their relative abundance and compared between Q7/Q7 and Q175/Q7 HD mice at 2 months (Glycerophospholipids, Supplementary Figures 6A,C; “Other Lipids”, Supplementary Figures 6B,D; *N* = 9 per genotype). Similar to results at 6 months the abundance of the many Glycerophospholipid subclasses did not differ by genotype in either fraction (PC, PE, SM, PS, PI, PG; Supplementary Figures 6A,C). However, several changes were identified among lipid subclasses of lower abundance (Table 2). In fraction 1, significant changes in 8 subclasses were found (Ceramides, neutral glycosphingolipids, MGDG, PA, PIP2, SQGQ, Sterols, and sphingolipid (phSM) (Table 2 and Supplementary Figure 6). The reduction in subclass of phSM in fraction 1 mirrors the change that was observed in a species of phSM at 6 months. In fraction 2, acylcarnitine (Acca) and diacylglyceride (DG) content were significantly decreased in Q175/Q7 compared to Q7/Q7 (Table 2 and Supplementary Figure 6D). Cholesterol ester was not detected at 2 months; Zymosterol ester was detected at 2 months but was not significantly changed (Supplementary Figure 6B).

**FIGURE 5:**
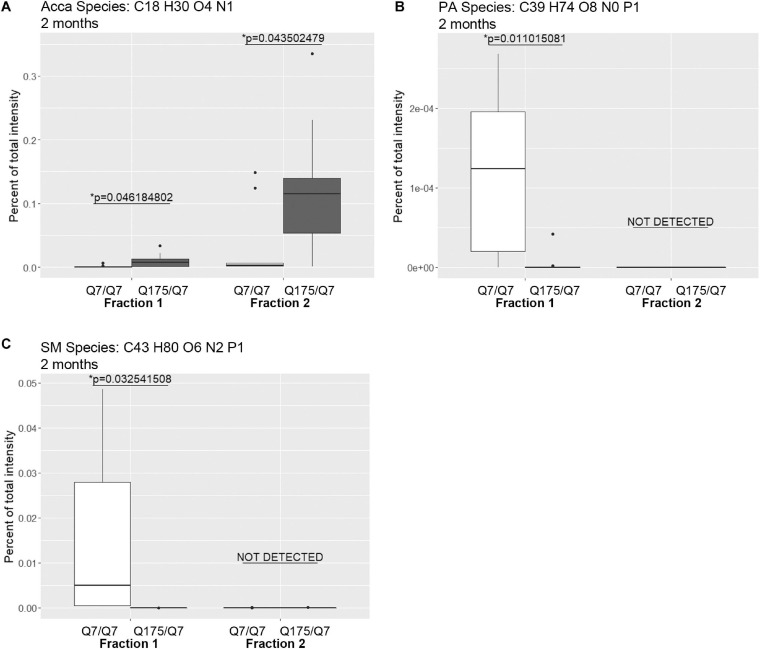
Levels of selected individual species of lipids detected by LC-MS/MS in fractions 1 and 2 of continuous density gradients of fractionated 2 months old Q175/Q7 and Q7/Q7 striata. Graphs show Tukey-style box plots where the lower and upper hinges correspond to the first and third quartiles (the 25th and 75th percentiles) as described in section “Materials and Methods.” Data beyond the end of the whiskers are called “outlying” points and are plotted individually. Outliers were not removed from statistical analysis. Changes at 2 months are shown for specific species of **(A)** Acyl Carnitine (Acca), **(B)** Phosphatidic acid (PA), and **(C)** Sphingomyelin (SM). Cholesteryl ester species was not detected by LC-MS/MS in the 2-month samples. All significant changes are shown in Supplementary Tables 1–8. Comparisons were made between genotype for each fraction (^∗^*p* < 0.05, unpaired *t*-test, *N* = 9 per group).

Looking at individual lipid species at 2 months also revealed numerous changes among lipids that were detected in both genotypes (Supplementary Table 3 for fraction 1 and Supplementary Table 6 for fraction 2). Many species of lipids were detected in Q7/Q7 but not detected in Q175/Q7 (Supplementary Table 4 for fraction 1 and Supplementary Table 7 for fraction 2). The reverse was also true: some lipids were detectible in Q175/Q7 that were not detected in Q7/Q7 (Supplementary Table 5 for fraction 1 and Supplementary Table 8 for fraction 2). One of the changes at 2 months was in a species of Acylcarnitine (Acca C18 H30 O4 N1) (Figure 5A) which was significantly higher in Q175/Q7 compared to Q7/Q7 for both fractions. This increase contrasts with results for Acca as a subclass which was significantly reduced in Q175/Q7 compared to Q7/Q7 in fraction 2 (Table 2). One possibility is that the decrease of several species of Acca drives a compensatory increase of this species of Acca or vice versus. A species of PA was significantly increased in fraction 1 (PA C39 H74 O8 N0 P1) (Figure 5B) but was not detected in fraction 2. A species of sphingomyelin (SM C43 H80 O6 N2 P1) had a similar profile to with a significant increase in fraction 1 but not detected in fraction 2 (Figure 5C). These results suggest real increases in these glycerophospholipid species and not just a shift that occurred with cell membrane redistribution in the gradient. Within the PIP2 group, a third PIP2 species #2 (C46 H88 O18 N0 P3) was detected in Q7/Q7 in fraction 1 but “not detected” in other fractions (Figure 4C). This same species of PIP2 was not changed at 6 months (Figure 4B).

Together, these data showed no change in the overall levels of glycerophospholipids that constitute the bulk of membranes in HD mice. However, distinct changes in individual species of PIPs occurred that reveal altered PI3 kinase signaling. Reduced levels of cardiolipin in both fractions from Q175/Q7 striata may have implications for mitochondrial function. Finally, a time dependent increase in cholesterol ester at 6 months in both fractions is indicative of dysfunctional intracellular cholesterol flux which may in turn impact cholesterol levels in membranes there-by altering the behavior of organelles in gradients.

### Electron Microscopy of Fractions 1 and 2 Reveals Differences in Subcellular Synaptic Components and Differences in Particle Size and Number Between 6-Month Old Q7/Q7 and Q175/Q7 HD Mice

**FIGURE 6:**
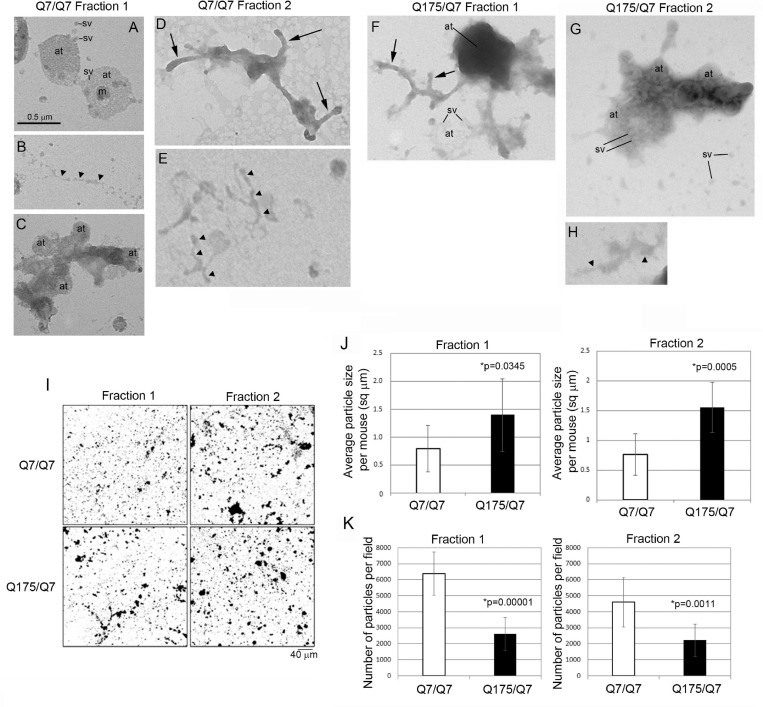
Electron microscopic analysis of subcellular morphology and particle sizes and numbers in fractions 1 and 2. **(A–H)** EM images of fractions 1 and 2. Specimens for EM analysis were prepared and analyzed as described in section “Materials and Methods.” Sv, synaptic vesicle; at, axon terminal; arrowheads, tubular-vesicular membranes that may be ER or endosomes **(B,E,H)**; arrows, preterminal axons and axon branches. Note that axon terminals (at) appear in groups in **(A,C,F)**, some are filled (axon terminals in **A**) or partly filled with vesicles (lower axon terminal in **F**) or overlap and appear electron dense (top axon terminal in **F**). Synaptic vesicles (sv) appear inside the axon terminals and scattered outside the terminals **(A,G)**. Note in **(G)** that axon terminals have irregular shapes, electron dense cytoplasm, and some larger irregular shaped vesicles. Scale bar in **A** = 0.5 μm and applies to all images. **(I–K)** Analysis of particle sizes and number in fractions 1 and 2. **(I)** shows representative electron microscope images of fractions 1 and 2 taken at 5,000× that were used for quantification. Scale bar = 40 μm and applies to all images. Data in **(J,K)** were obtained using ImageJ. Threshold was set for images on automatic settings using Renyi entropy method. Particles were excluded that were only 1 pixel. Students *t*-test was performed on *N* = 9 animals for each genotype. Bar graph in **(J)** shows mean particle size ± SD per mouse for Q7/Q7 and Q175/Q7 samples for fractions 1 and 2 (^∗^*p* < 0.05, unpaired *t*-test, *N* = 9 mice, 7–45 images per mouse). There are significantly larger particles in Q175/Q7 samples compared to Q7/Q7 in fraction 1 and fraction 2. Bar graph in **(K)** shows number of particles ± SD per field for Q7/Q7 and Q175/Q7 samples for fractions 1 and 3 (*N* = 9 mice, 7–45 image per mouse). There are significantly fewer particles in Q175/Q7 samples compared to Q7/Q7 in fraction 1 and fraction 2.

The results above showed that the HD mutation affected the subcellular distribution of membrane associated proteins and lipids in light fractions 1 and 2. To verify the ultrastructural constituents of fractions 1 and 2, electron microscopic analysis was performed. Drops of each fraction were placed on formvar grids and stained with uranyl acetate and lead citrate. Analysis at 30,000 X revealed microscopic fields that were heterogeneous in the type and density of subcellular structures and included those characteristic of synaptic enrichment as previously described by Whittaker et al. (1964). Fractions 1 and 2 of Q7/Q7 and Q175/Q7 mice (Figures 6A–H) contained areas with scattered synaptic vesicles and axon terminals that were vesicle filled (Figures 6A,G) or partly devoid of vesicles (lower axon terminal in Figure 6F). The synaptic vesicles were 40–50 nm in diameter and mostly lucent. Some axon terminals had mitochondria (Figure 6A). The plasma membrane encircling the axon terminals was more electron dense than the vesicle membranes and appeared interrupted (best seen in upper axon terminal of Figure 6A). The axon terminals appeared singly or in clusters (Figures 6 A,C,F,G). The axon terminals were mainly round or ovoid shaped in Q7/Q7 fraction 1 and 2 and in Q175/Q7 fraction 1. In Q175 fraction 2, terminals had irregular shapes, electron dense cytoplasm, and some larger irregular shaped vesicles (Figure 6G). Some axon terminals that were seen in clusters appeared proximal to tubular shaped structures with short branches that we interpreted to be pre-terminal axons. These pre-terminal axons which were also seen separate from axon terminals, appeared mostly in fraction 2 of Q7/Q7 preparations and fraction 1 of Q175/Q7 samples (Figures 6D,F). Present but more scattered in both fractions were larger round clear vesicles (100–200 nm) consistent with the size of microsomes that are derived from ER or lipid droplets. Also present were electron lucent irregular-shaped tubulovesicular membranes (Figures 6B,E,H) that may belong to ER or endosomes.

Since there was heterogeneity across microscopic fields and some difference in organelle distribution in fractions 1 and 2 between Q7/Q7 and Q175/Q7 HD samples, we sought to determine whether the overall density and size distribution of subcellular structures was the same between Q7/Q7 and Q175/Q7 HD mice. EM analysis was performed on 500x images (Figure 6I) to examine particle size and number in fractions 1 and 2 of the 6-month-old Q7/Q7 and Q175/Q7 HD mice as described in Methods. Results showed a significant difference in particle size and number of particles in fractions 1 and 2 between Q7/Q7 and Q175/Q7 HD (Figures 6J,K). Particle size was significantly increased in both fractions 1 and 2 prepared from Q175/Q7 HD compared to Q7/Q7 mice (Figure 6J). Conversely, particle number was reduced in both fractions 1 and 2 prepared from Q175/Q7 HD compared to Q7/Q7 (Figure 6K).

### Analysis of Human Putamen Shows Alterations in Subcellular Distribution of Some Synaptic Proteins

**FIGURE 7:**
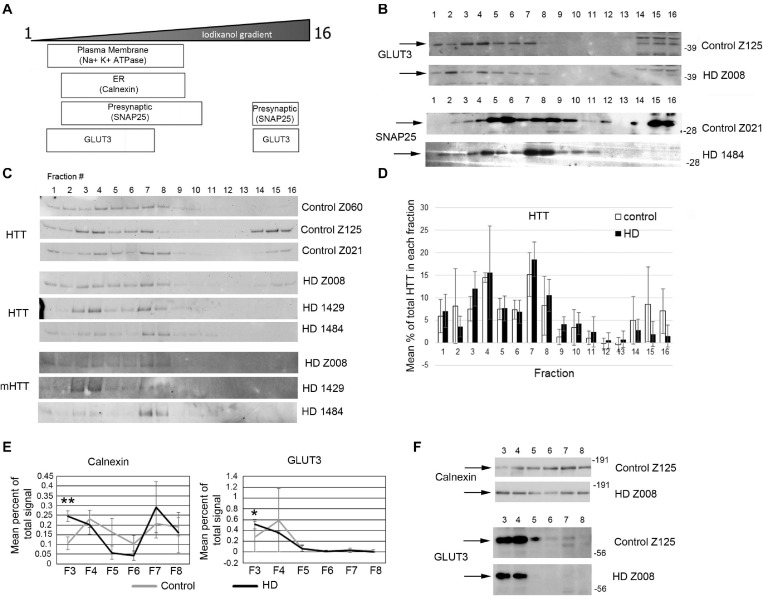
Subcellular fractionation by density gradient ultracentrifugation of human putamen. Dorsal putamen was fractionated as described in section “Materials and Methods.” Fractions were assessed by SDS-PAGE and western blot analysis using different cell compartment markers. Fractions 1–16 were removed from top to bottom. Equal volumes of each fraction were loaded per lane. **(A)** Diagram at left shows approximate distribution of proteins representative of different subcellular compartments in the control putamen. **(B)** Shown are representative images of western blots detecting GLUT3 and SNAP25 in control and HD putamen. **(C)** Shown are WB images of Huntingtin (HTT) and mutant HTT (mHTT) signals in 16 fractions from all human putamen samples. mHTT was detected with 1C2 which recognizes the expanded polyQ region in mutant HTT but not HTT in control samples. **(D)** Bar graph shows mean percent ± SD of HTT signal obtained with antibody Ab1 in control and HD human putamen in each of 16 fractions as a percent of total HTT signal summed across all fractions. There was no significant difference in HTT signal between control and HD in any of the fractions (*N* = 3). **(E)** Fractions 3–8 were analyzed in pairs and run on the same gel to eliminate variability due to different runs. These fractions were chosen because most proteins tested had peaks in this range. Seven probes were analyzed by western blot for each gel (Calnexin, GLUT3, Na+/K+ ATPase, NMDAR 2b, PSD95, SNAP25, and XK). Band intensity for each fraction was standardized to sum of intensities for bands in fractions 3–8. Line graphs show mean percent of total signal ± SD obtained in fractions 3–8 for each fraction (*N* = 3). There was a significant difference in percent of total signal in fractions 3–8 in fraction 3 for Calnexin (**p = 0.0052, *N* = 3, unpaired *t*-test for all analyses) and GLUT3 (*p = 0.047). **(F)** Representative images of western blots for Calnexin and GLUT3 in control and HD putamen related to the data shown. See Supplementary Figure 5 for other line graphs.

Previous study has shown successful isolation of synaptosome fractions from postmortem fresh brain tissue of different species using sucrose for separation (Shinagawa et al., 1963; Whittaker et al., 1964; Garey et al., 1974; Sapp et al., 2020). To determine whether frozen postmortem tissue could be assessed using iodixanol gradient fractionation and if HD mutation affected the subcellular distribution of proteins in the synaptic compartment, we examined samples of human putamen from 3 controls to 3 HD postmortem brains (Supplementary Table 9). Relative distribution of subcellular markers after fractionationis similar to that seen in mice although SNAP25 separated to higher fractions than in mice in human putamen (Figures 7A,B). Similar to the mouse western blot results, no difference in distribution of wild-type and mutant Huntingtin in the fractions between control and HD putamen was observed (Figures 7C,D). There was a significant difference between control and HD in fraction 3 for Calnexin (*p* = 0.0052, *N* = 3, unpaired *t*-test) and GLUT3 (*p* = 0.047, *N* = 3, unpaired *t*-test) (Figures 7E,F). There was no significant difference in subcellular distribution between control and HD for Na^+^/K^+^ ATPase, NMDAR 2b, SNAP25, VGLUT1, and PSD95 (Supplementary Figure 7). These results show that human postmortem tissue can be used for analysis of subcellular fractions using iodixanol and that the HD mutation alters the distribution of some synaptic proteins.

## Discussion

Altered function of striatal neurons including synaptic interactions occurs early in HD pathogenesis. Here we used an HD mouse model (Q175/Q7) and human HD postmortem brain to address if there is a change in the subcellular distribution of membrane associated proteins and lipids in the HD striatum. We found a change in the relative distribution of wild-type and mutant Huntingtin in a single fraction (fraction 4) but a change in the distribution of five membrane proteins (intrinsic or membrane-associated) in synaptic fractions generated by iodixanol separation of Q175/Q7 HD mice versus Q7/Q7 mouse brain lysates providing evidence to support synaptic changes in the HD mice. Lipidomic analysis of these fractions identified changed distribution of cholesterol ester, cardiolipin, PIP2 and numerous other lipids. EM analysis confirmed the presence of synaptic profiles and revealed abnormal morphology of some axon terminals and vesicles in Q175/Q7 HD mice. Together, these data suggest a physical and/or structural change in membrane or organelle integrity or a redistribution of several proteins among membrane compartments which becomes evident after disruption by homogenization and fractionation.

Mounting evidence from studies using many different approaches have shown that changes in synaptic function are the basis for early cognitive and psychiatric symptoms in HD (Giralt et al., 2012) and account for deficits in motor control including initiation and execution of movement (Reiner and Deng, 2018) and involve changes in pre- and postsynaptic sites (Nithianantharajah and Hannan, 2013). Reduced glutamate release in HD has been ascribed to impaired synaptic release due to physical association of mutant Huntingtin with synaptic vesicles (Li et al., 2003). Impaired glutamate release has also been reported in YAC128 transgenic mice (Joshi et al., 2009) and in synaptosomes from Q140/Q140 knock-in mice (Valencia et al., 2013). Altered GABA transmission occurs in R6/2 mice (Cepeda et al., 2004). Mutant Huntingtin expression altered functions of Synapsin 1 when targeted to the synapse in transgenic mice (Xu et al., 2013), reduces transcription of Synaptic Vesicle Protein 2C in N171-82Q transgenic mice (Peng et al., 2018) and affected interactions with Syntaxin 1A (Kaltenbach et al., 2007). Differences were found in levels of pre- and postsynaptic proteins in Q50/Q7, Q140/Q7, Q175/Q7, and Q140/Q140 HD mouse brain synaptosomes compared to wild-type including SCN4B, GABAa1, calmodulin, VGlut1, VGlut2, SNAP25, and PSD95 (Valencia et al., 2013; Sapp et al., 2020). Significant loss of the postsynaptic signaling proteins, PDE10a and DARPP32 was reported by us and others (Beaumont et al., 2016; Hosp et al., 2017; Skotte et al., 2018; Sapp et al., 2020). Here using a biochemical analysis of subcellular fractions separated by iodixanol (OptiPrep) we found no overall change in Huntingtin distribution except in fraction 4 but an altered distribution of 5 proteins within synaptic fractions of Q175/Q7 HD mice. They include plasma membrane proteins (Na^+^/K^+^ ATPase and GLUT3), presynaptic (vGlut1) and postsynaptic proteins (PSD95 and NMDAR 2B). A shift in presynaptic SNAP25 was nearly significant (*p* = 0.0527) as was another post synaptic receptor protein, AMPA Glu1A (*p* = 0.06). The results further support the idea that both pre- and postsynaptic features are affected in HD.

Four of the five proteins with aberrant subcellular distribution in HD mice are trans membrane proteins bearing membrane spanning domains. This finding suggests that there is (1) a change in membrane architecture which transforms biochemical behavior or (2) modified targeting/distribution among cellular membranes. In HD mouse models, redistribution of NMDA 2b between postsynaptic and extra-synaptic sites on plasma membranes has been demonstrated using electrophysiology and immunofluorescent readouts (Fan et al., 2007; Milnerwood et al., 2012). The shift we observed in biochemical gradients for NMDA 2b from fraction 2 in Q7/Q7 mice to fraction 1 in Q175/Q7 preparations may reflect the shift from postsynaptic to extra-synaptic membranes described by Raymond and colleagues. The extra-synaptic shift they described for NMDA 2b is regulated by palmitoylation at cysteine cluster II (Kang et al., 2019). Palmitoylation occurs dynamically for many synaptic proteins (Kang et al., 2008) and can regulate their trafficking (Kang et al., 2004). Interestingly, PSD95 was shifted and SNAP25 was trending toward significance (*p* = 0.0527) and both are also palmitoylated. Thus, palmitoylation catalyzed by the Huntingtin interactor proteins HIP14 (zDHHC17) or HIP14L (zDHHC13) may account for the biochemical redistribution we observe. In fact, palmitoylation has been reported for vGlut1 (Zareba-Koziol et al., 2018), Na^+^/K^+^ ATPase (Plain et al., 2020), and GLUT3 (Stapel et al., 2019), AMPAR and GABAR (Zareba-Koziol et al., 2018).

Abnormal receptor mediated endocytosis and recycling could explain the redistributed proteins seen in our study in HD mice. Wild-type Huntingtin interacts with Rab11 (Li et al., 2008) and mutant Huntingtin disrupts Rab11 dependent recycling of transferrin, GLUT3, and EAAC1 receptors (Li et al., 2010). Huntingtin also interacts with proteins involved in endocytosis including AP2 (Faber et al., 1998), and dynamin (El-Daher et al., 2015). Depolarization-dependent endocytosis is a specific form of endocytosis at synapses and is used to retrieve membrane and proteins after synaptic vesicle release [reviewed by Lou (2018)] and utilizes proteins known to interact with and be affected by mutant Huntingtin such as Synapsin 1 (Valencia et al., 2013; Xu et al., 2013). One possibility is that depolarization dependent recycling is changed in HD creating altered distribution of membrane proteins between the plasma membrane and internal vesicles.

Remarkably, frozen samples of human postmortem brain of control and HD individuals could be separated by iodixanol (OptiPrep) to reveal a relative distribution of subcellular markers and of Huntingtin similar to that in mice, although the synaptic proteins PSD95 and SNAP 25 were separated to higher fractions (3–6) than in mice in both control and HD putamen. Since membranous compartments may change with freezing and thawing, one would not expect the frozen postmortem human tissue to fractionate identically to freshly dissected mouse tissue. Only GLUT3 and calnexin were changed in human HD putamen with both proteins being increased compared to control in fraction 3. The small number of cases available for analysis (*N* = 3) may account for the limited number of proteins affected. The transmembrane protein GLUT3 which was also changed in Q175/Q7 HD mice striatum (decreased compared to wild-type in fraction 2) is noteworthy since as noted above this protein is the major glucose transporter in neurons and its recycling is impaired in HD due to reduced Rab11 activity (McClory et al., 2014).

Lipidomics of synaptic fractions 1 and 2 from wild-type and HD mice were compatible with the biochemical protein data in revealing significant lipid changes in these fractions. For example, there was increased cholesterol ester content in Q175/Q7 HD mice synaptic fractions. Cholesterol ester is a storage form of cholesterol created by modification with an acyl chain by esterification at the OH group of cholesterol (by Acat-1 and fatty acyl co-A). Cholesterol ester is moved within cells via lipid transfer proteins such as cholesterol ester transport protein (CETP) and is stored almost exclusively in lipid droplets (Wong et al., 2019). A change in the load of cholesterol ester may contribute to altered membrane stiffness by sequestering cholesterol away from plasma membranes. Cholesterol is an important component of plasma membranes and is required for synaptogenesis (Mauch et al., 2001), normal learning (Xu et al., 1998), and axon regeneration (de Chaves et al., 1997). Changes in cholesterol levels are reported to affect both presynaptic events (Thiele et al., 2000; Dason et al., 2010, 2014; Linetti et al., 2010), and postsynaptic architecture including localization of NMDAR (Frank et al., 2004, 2008) and AMPARs (Hering et al., 2003; Renner et al., 2009; Martin et al., 2014). Augmenting cholesterol in brain improved electrophysiology readouts in R6/2 HD mice which expresses mutant Huntingtin exon 1 including normalizing both membrane capacitance and inhibitory postsynaptic current (IPSC) frequencies in striatal medium spiny neurons (Valenza et al., 2015a). Although behavior deficits were not rescued, some cognitive dysfunction was improved in R6/2 mice treated with direct delivery of cholesterol to brain and levels of some synaptic proteins were preserved (Valenza et al., 2015a).

There is evidence that the HD mutation affects cholesterol levels. We and others have reported decreased levels of total cholesterol in Q140/Q140 HD mouse primary neurons (Ritch et al., 2012), synaptosomes (Valenza et al., 2010) and brain (Valenza et al., 2007). It is noteworthy that the distribution of cholesterol enriched lipid rafts which mediate signaling at plasma membranes is altered in HD (Valencia et al., 2010). In human plasma 24S-hydroxycholesterol (24S-OHC) a form of cholesterol modified to allow efflux across the blood brain barrier is reduced (Leoni et al., 2008, 2013). These findings have led to mechanistic studies supporting the hypothesis that cholesterol synthesis is reduced in HD (Valenza et al., 2005). However, cholesterol homeostasis is complex and increases in total cholesterol have also been reported using colorimetric assays (Trushina et al., 2006; Del Toro et al., 2010). Of note, knockout mice null for expression of CYP4A1, the enzyme which creates 24SOHC displays normal levels of cholesterol indicating tight feedback regulation: reduced efflux results in decreased production to maintain cholesterol homeostasis (Lund et al., 2003). Our data showing increased cholesterol ester in fractions containing synaptic elements suggest that in addition to reduced cholesterol production, altered redistribution within cells or flux may also play a role in changing membrane function. Indeed, changes in cholesterol flux affect membrane ordering and structure, ultimately impacting synaptic function including glutamate release (Petrov et al., 2020).

Other changes in lipid levels that we observed in HD mouse synaptic fractions may give insights into HD pathology. Cardiolipin was reduced in fraction 1 of Q175/Q7 HD mouse striatum at 6 months. We showed that wild-type and mutant Huntingtin have a strong association with cardiolipin *in vitro* (Kegel et al., 2009). Cardiolipin is enriched in mitochondrial membranes and its reduction in mitochondria of HD synapses could affect bioenergetics, a problem known to occur in HD. Cardiolipin is usually found on inner mitochondrial membranes (Chicco and Sparagna, 2007) but with apoptotic stimulation can also be located at the interface of inner and outer mitochondrial membranes (Gonzalvez et al., 2008). Mutant Huntingtin has been found on the outer mitochondrial membranes and can affect cytochrome c release and apoptosis (Choo et al., 2004; Orr et al., 2008). However, recently Huntingtin was detected in the intermembrane space of mitochondria and therefore could potentially be in contact with cardiolipin (Yablonska et al., 2019).

An increase in Acyl carnitine occurred in both fractions 1 and 2 of Q175/Q7 from striatum for one species and was higher as a subclass in fraction 2 at 2 months. DAG was also higher as a subclass in fraction 2 at 2 months. Acyl carnitine and DAG are precursor energy metabolites. Acyl carnitine is imported to mitochondria for conversion to ATP. The increased levels of Acyl carnitine could indicate reduced transport by lipid import/transport proteins into mitochondria or a loss of mitochondria both of which would result in reduced energy metabolism.

We found a change in a less abundant PIP2, most likely PI(3,4)P2 or PI(3,5)P2. Wild-type and mutant Huntingtin bind all PIP2s and mutant Huntingtin has altered binding to PIP3 (not detected here) and PIPs with lower affinity (Kegel et al., 2005, 2009). Mutant Huntingtin could affect the creation of PI(3,4)P2 and/or PI(3,5)P2 which is catalyzed by PI 3-kinase family members on cellular membranes including plasma membranes and autophagic vesicles. Alternatively, mutant Huntingtin could interfere with effector molecules that bind PIP2s. PIP2s are particularly important in synaptic compartments where PI(4,5)P2 controls endocytosis of synaptic vesicles in axon terminals [reviewed by Koch and Holt (2012)] and where PI(3,5)P2 has been shown to control localization of AMPA receptors post-synaptically (McCartney et al., 2014; Kim et al., 2017). Altered levels of these PIP lipid signaling molecules direct changes in membrane trafficking through Rab proteins (Tan et al., 2015) and changes in actin dynamics through Rac1. We have shown that a Rac1/PI 3-kinase/Huntingtin/alpha-actinin-2 protein complex forms incorrectly using lysate from wild-type and knock-in Q140/Q140 mouse striatum (Tousley et al., 2019b).

Consistent with our reported changes in PA in Q140/Q140 HD knock-in mice at 11 months (Vodicka et al., 2015), we saw changes in the subclass PA with decreased PA in Q175/Q7 fraction 1; some species of PA were undetectable in mutant cells compared to Q7/Q7. PA is a building block for other phospholipids but also a signaling molecule itself.

There was no change in the distribution pattern of wild-type and mutant Huntingtin in subcellular fractions, although a significant decrease in mutant Huntingtin compared to wild-type occurred in fraction 4 of Q175/Q7 mice compared to Q7/Q7 mice. These data showing normal targeting of mutant Huntingtin are consistent with earlier studies (Aronin et al., 1995; Sharp et al., 1995; Velier et al., 1998; Kim et al., 1999). The fact that mutant Huntingtin fractionates normally in fractions 1 and 2 but several other proteins do not implies a change in mutant Huntingtin function. Mutant Huntingtin may create problems by interacting as a soluble monomer or as an aggregate in the synapse (Li and Conforti, 2013; Sapp et al., 2020). As a soluble monomer, mutant Huntingtin may change the function of numerous interactor proteins [reviewed by Wanker et al. (2019)]. Mutant Huntingtin also changes the function of two interactor proteins, HIP14 and HIP14L, which are palmitoyl acyltransferases that can impact synaptic function (Kang et al., 2019). Conversely, aggregation has been shown to drastically alter the proteome of R6/2 mice which may have profound consequences for function (Hosp et al., 2017). In addition, mutant Huntingtin may also indirectly affect synaptic function by changing transcription of synaptic genes (Langfelder et al., 2016) or have pleiomorphic effects. For instance, alpha-actinin-2, which is an actin bundling protein, interacts with Huntingtin (Tousley et al., 2019a) and also interacts with NMDA 2b in spines (Wyszynski et al., 1997). Alpha-actinin-2 is also transcriptionally downregulated in HD models (Langfelder et al., 2016) which may create instability in spines and impact NMDA 2B receptors distribution. Mutant Huntingtin also alters transcription of fatty acid and cholesterol metabolism in neurons through direct effects on SREB in astrocytes (Valenza et al., 2015b).

Transmission electron microscopy confirmed our biochemical findings that fractions 1 and 2 were enriched in synaptic profiles. These observations which were made in fixed droplets (with no embedding or thin sectioning) of fractions 1 and 2 in Q7/Q7 and Q175/Q7 mice showed synaptic vesicles in isolation and vesicle filled axon terminals including some that contained intact mitochondria. The presence of presynaptic terminals agrees with detection of SNAP25 by western blot in fractions 1 and 2 of the Q7/Q7 and Q175/Q7 and mouse striatum. These features are among those also seen in synaptosome fractions separated in 0.32 sucrose buffer. Synaptosomes fractionated by sucrose gradient are enriched for presynaptic profiles with some profiles having attached postsynaptic densities as we and others have seen by EM analysis (De Robertis et al., 1961; Garey et al., 1974; Sapp et al., 2020). In contrast, it was difficult to find evidence of a post-synaptic density on the axon terminal membranes in the iodixanol separated fractions by EM despite detection of PSD95 in fractions 1–2 by western blot. These results suggest that these membranes and densities are still present but not recognizable. We did see interruptions in the continuity of the plasma membranes of some axon terminals which could relate to an altered distribution of lipids including cholesterol. The increased prevalence of preterminal axons in fraction 1 of Q175 compared to Q7 is unclear. The presence of multilamellar processes (myelinated axons) sometimes noted at the EM level in synaptosomes isolated by a sucrose gradient (Petrushka and Giuditta, 1959; Shinagawa et al., 1963) were not present in our iodixanol separated fractions 1 and 2. Marked heterogeneity and polymorphic profiles have been noted in synaptosome preparations obtained using sucrose (Petrushka and Giuditta, 1959; Shinagawa et al., 1963; Whittaker et al., 1964). Our method for preparing fractions for EM analysis and the ultrastructural appearance observed was most similar to the images and descriptions of Whittaker et al. who also applied fixed droplets of non-embedded fractions to a coated grid (Whittaker et al., 1964).

Ultrastructural analysis at low magnification revealed increased size and decreased particle number in Q175/Q7 fraction 1 and 2 compared to Q7/Q7 fractions. While the significance of these findings from isolated fractions is unclear, there is evidence that a depletion of synapses occurs in the intact striatum of Q140/Q7 HD mice (Deng et al., 2013). It is possible that during preparation of the fractions in our study homogenization weakened structural integrity to give rise to the altered axon terminal and vesicle morphology we observed. Huntingtin has been implicated in actin morphology directly through interactions with actin (Angeli et al., 2010) and interactions with actin binding proteins such as profilin, cofilin, alpha-actinins (Culver et al., 2012; Shirasaki et al., 2012; Tourette et al., 2014; Tousley et al., 2019a) and mutant Huntingtin can exert negative effects through ROCK (Shao et al., 2008), Rac1 (Tousley et al., 2019b) and potentially other interacting proteins such as HIP1 and GIT1 (Harjes and Wanker, 2003; Goehler et al., 2004). Alternatively, failure of vesicles to bud due to impaired Rab11 dependent trafficking in the presence of mutant Huntingtin (Li et al., 2009) may account for the change in distribution in size of measured particles.

In summary, the work presented here using subcellular fraction methods suggests that the HD mutation alters the physical distribution of proteins and the levels of specific lipids important to membrane functions at the synapse. Multiple membrane compartments may be impaired including the plasma membrane, endosomes, and mitochondrial membrane. The impairment of membrane functions fits with previously identified abnormal protein interactions by mutant Huntingtin that affect extra-synaptic redistribution of synaptic proteins, endosome recycling, the organization of lipid rafts, and mitochondria membrane functions.

## Data Availability Statement

The original contributions presented in the study are included in the article/Supplementary Material, further inquiries can be directed to the corresponding author/s.

## Ethics Statement

The animal study was reviewed and approved by the MGH Subcommittee on Research Animal Care (SRAC)-OLAW #2004N000248.

## Author Contributions

MI and CS performed the experiments and data analysis, and wrote the manuscript. ES performed the experiments and edited the manuscript. EJ performed electron microscopy. CM performed the experiments. XL contributed to study design and preliminary experiments. MD and KK-G contributed to study design and oversight and wrote and edited manuscript. All authors contributed to the article and approved the submitted version.

## Conflict of Interest

KK-G spouse owns less than 0.1% stock in the following companies: Bristol-Myers Squibb Company, Cisco Systems, Inc., GE Healthcare Life Sciences, Generex Biotechnology Corporation, GlaxoSmithKline, Metabolix Bioplastics, Nanogen, Inc., Nanometrics Inc., Repligen, and StemCells, Inc. The remaining authors declare that the research was conducted in the absence of any commercial or financial relationships that could be construed as a potential conflict of interest.
