# Supplementary material for: Disposition of Proteins and Lipids in Synaptic Membrane Compartments Is Altered in Q175/Q7 Huntington’s Disease Mouse Striatum
Source: Front Synaptic Neurosci. 2021 Mar 18;13:618391. doi: 10.3389/fnsyn.2021.618391 (PMC8013775; doi:10.3389/fnsyn.2021.618391)
Supplement: Supplementary file 1 [file Data_Sheet_1.docx]

**Supplementary Tables and Figures**

**Supplementary Table 1 Altered Lipid species at 6 months in Fraction1**

Values in columns 5-8 represent total class intensities standardized to total intensity for each sample.

Red; decreased in HD

Green; increased in HD

**Supplementary Table 2 Altered lipid species at 6 months in Fraction 2**

Values in columns 5-8 represent total class intensities standardized to total intensity for each sample.

Red; decreased in HD

Green; increased in HD

**Supplementary Table 3 Altered Lipid species at 2 months in Fraction 1**

Values in columns 5-8 represent total class intensities standardized to total intensity for each sample.

Red; decreased in HD

Green; increased in HD

**Supplementary Table 4 Lipid species not detectable in Q175/Q7 at 2 months in Fraction 1**

Values in columns 5-8 represent total class intensities standardized to total intensity for each sample.

Red; decreased in HD

Green; increased in HD

**Supplementary Table 5 Lipid species not detectable in Q7/Q7 at 2 months in Fraction 1**

Values in columns 5-8 represent total class intensities standardized to total intensity for each sample.

Red; decreased in HD

Green; increased in HD

**Supplementary Table 6 Altered Lipid Species at 2 months in Fraction 2**

Values in columns 5-8 represent total class intensities standardized to total intensity for each sample.

Red; decreased in HD

Green; increased in HD

**Supplementary Table 7 Lipid species not detectable in Q175/Q7 at 2 months in Fraction 2**

Values in columns 5-8 represent total class intensities standardized to total intensity for each sample.

Red; decreased in HD

Green; increased in HD

**Supplementary Table 8 Lipid species Not Detectable in Q7/Q7 at 2 months in Fraction 2**

Values in columns 5-8 represent total class intensities standardized to total intensity for each sample.

Red; decreased in HD

Green; increased in HD

**Supplementary Table 9. Human brain information**

| **Code** | **Genotype** | **Age** | **Sex** | **PMI (hrs)** |
| --- | --- | --- | --- | --- |
| **Z060** | Control | 76 | F | 12.75 |
| **Z125** | Control | 64 | M | 17 |
| **Z021** | Control | 68 | F | 11 |
| **Z008** | HD | 61 | F | 11 |
| **1429** | HD | 72 | M | 19 |
| **1484** | HD | 78 | F | 13 |

PMI = Post mortem interval; CAG repeat data not available, cases were verified as HD by the presence of mutant huntingtin in western blot assay

**
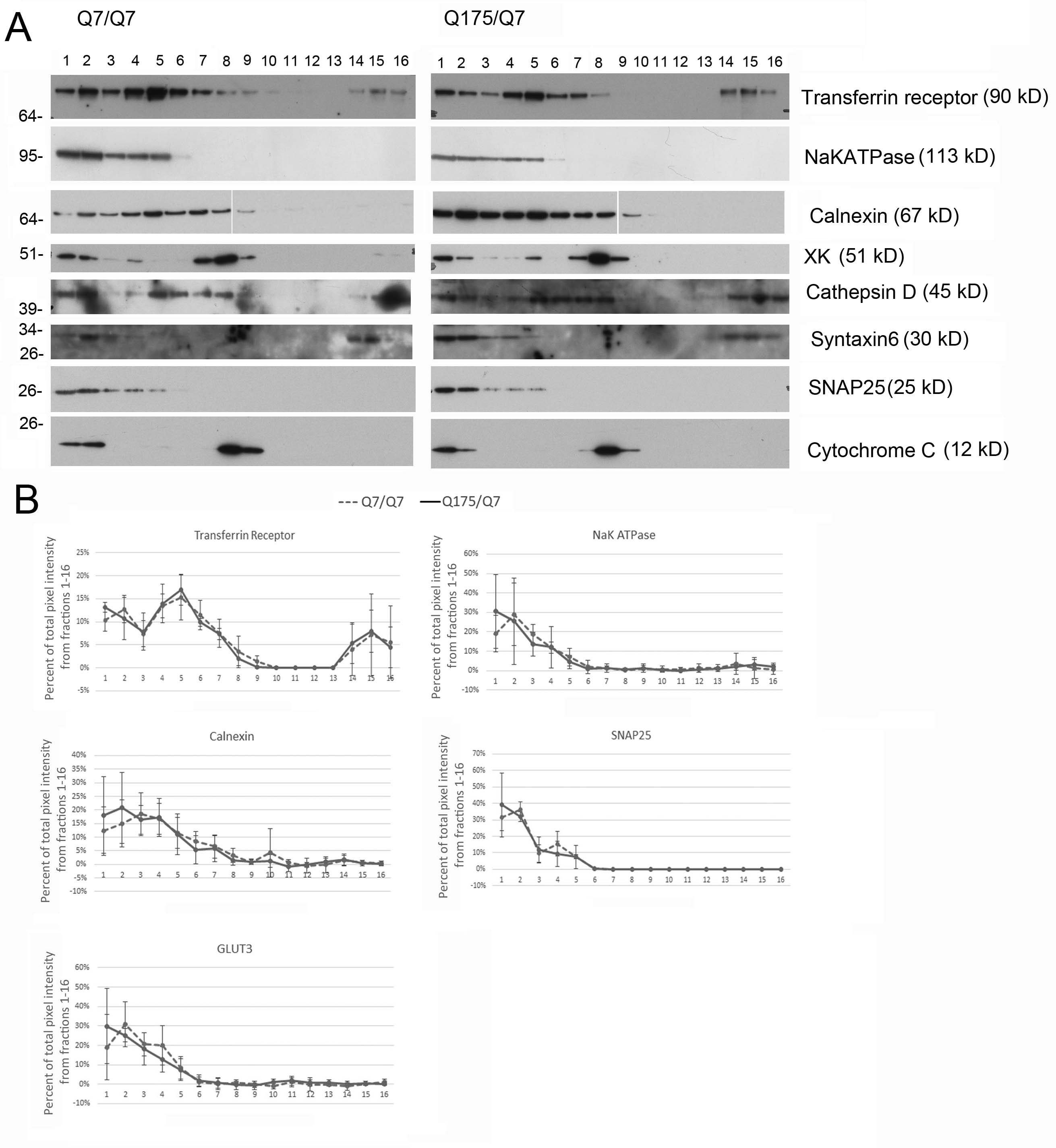
**

**Supplementary Figure 1. Subcellular distribution of different proteins using Optiprep gradients for the separation.** Postnuclear supernatants from fresh striatal homogenates were under-laid and separated on 10-35% continuous Optiprep gradients by ultracentrifugation in a SW41 swing bucket rotor for 2.5 hours at 37,000 rpm (100,000xg) at 4 degrees C**.** Western blots of 0.75 ml fractions (Fractions 1-16) were loaded by equal volume onto two SDS-PAGE gels (4-12% Bis-Tris). A. Examples are shown of blots probed with antibodies against proteins that are known to be enriched in different subcellular compartments: SNAP25 (pre-synaptic vesicles), Syntaxin6 (TGN and TGN-derived small vesicles en route to PM), Transferrin receptor (PM and recycling compartment), Na+/K+ ATPase (PM), calnexin (endoplasmic reticulum (ER), Cathepsin D (lysosome), and cytochrome C (mitochondria). B. Graphs of average percent of total pixel intensity for fractions 1-16 are shown for N=3-5 mice.

**
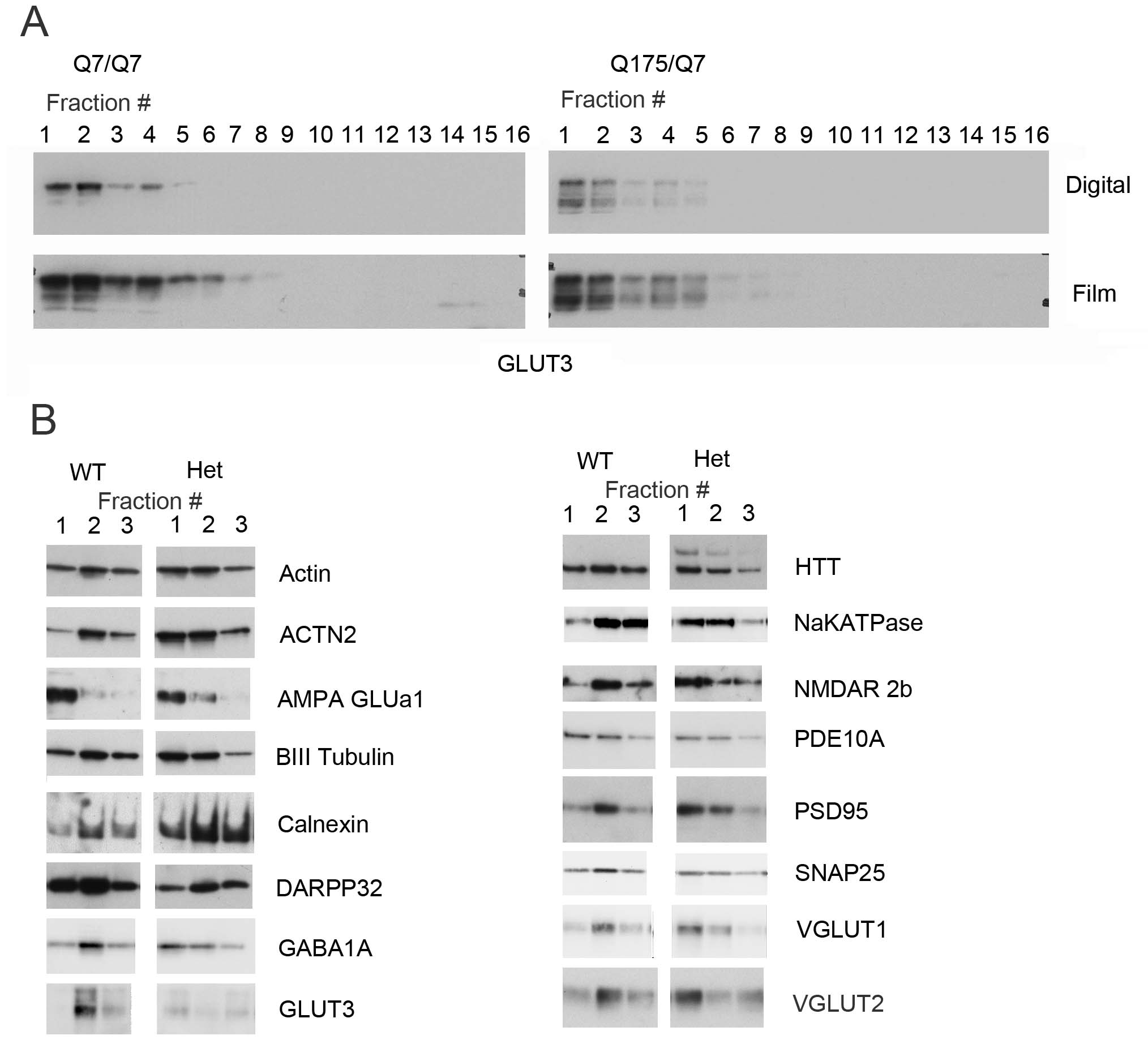
**

**Supplementary Figure 2. A.** Shown are representative images taken with digital camera and scanned film for GLUT3. **B.** Representative images of fractions 1, 2, and 3 taken either with digital camera or scanned film for the data graphed in Figure 2.


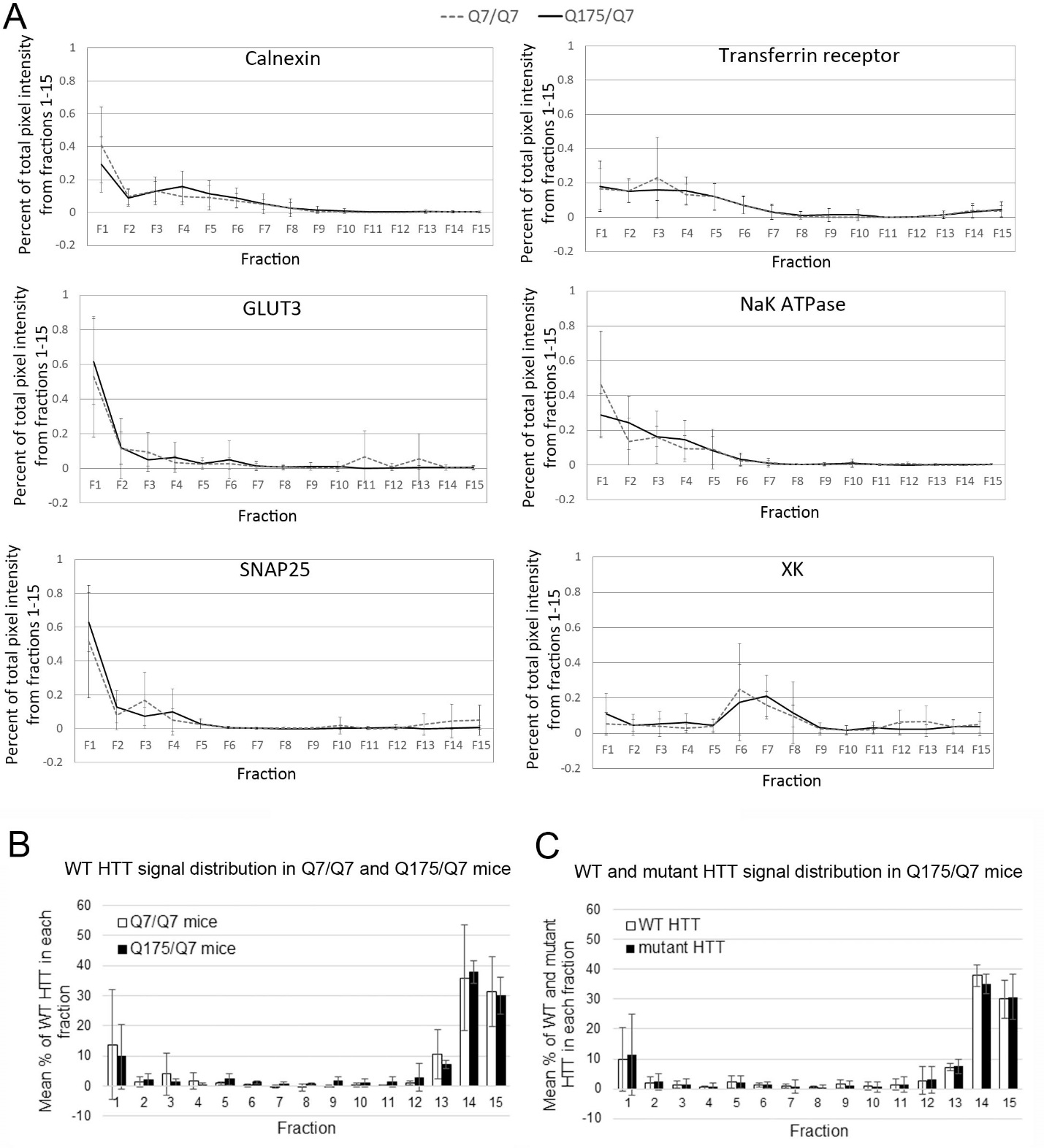


**Supplementary Figure 3. Subcellular fractionation by density gradient ultracentrifugation of 2-month old mouse striatum.** **A.** Line graphs show mean percent of total signal ± SD obtained in fractions 1-15 for each fraction. There was no significant difference between Q7/Q7 and Q175/Q7 in any of the fractions for Calnexin (N=9), Transferrin receptor (Q7/Q7 N=8, Q175/Q7 N=9), GLUT3 (N=8), NaK ATPase (N=9), SNAP25 (Q7/Q7 N=8, Q175/Q7 N=6) or XK (N=9). **B.** Bar graph shows mean ± SD WT Huntingtin (HTT) signal as a percent of total WT HTT signal in each of 15 fractions summed across all fractions in Q7/Q7 and Q175/Q7 2-month old mice. There was no significant difference in WT HTT signal between Q7/Q7 and Q175/Q7 mice for any of the fractions (unpaired t test, N=3). **C**. Bar graph shows percent of WT and mutant HTT signal in each of 15 fractions as a percent of total WT and mutant HTT signal summed across all fractions in Q175/Q7 2-month old mice. There was no significant difference between WT and mutant HTT signal in Q175/Q7 mice for any of the fractions (unpaired t test, N=3).


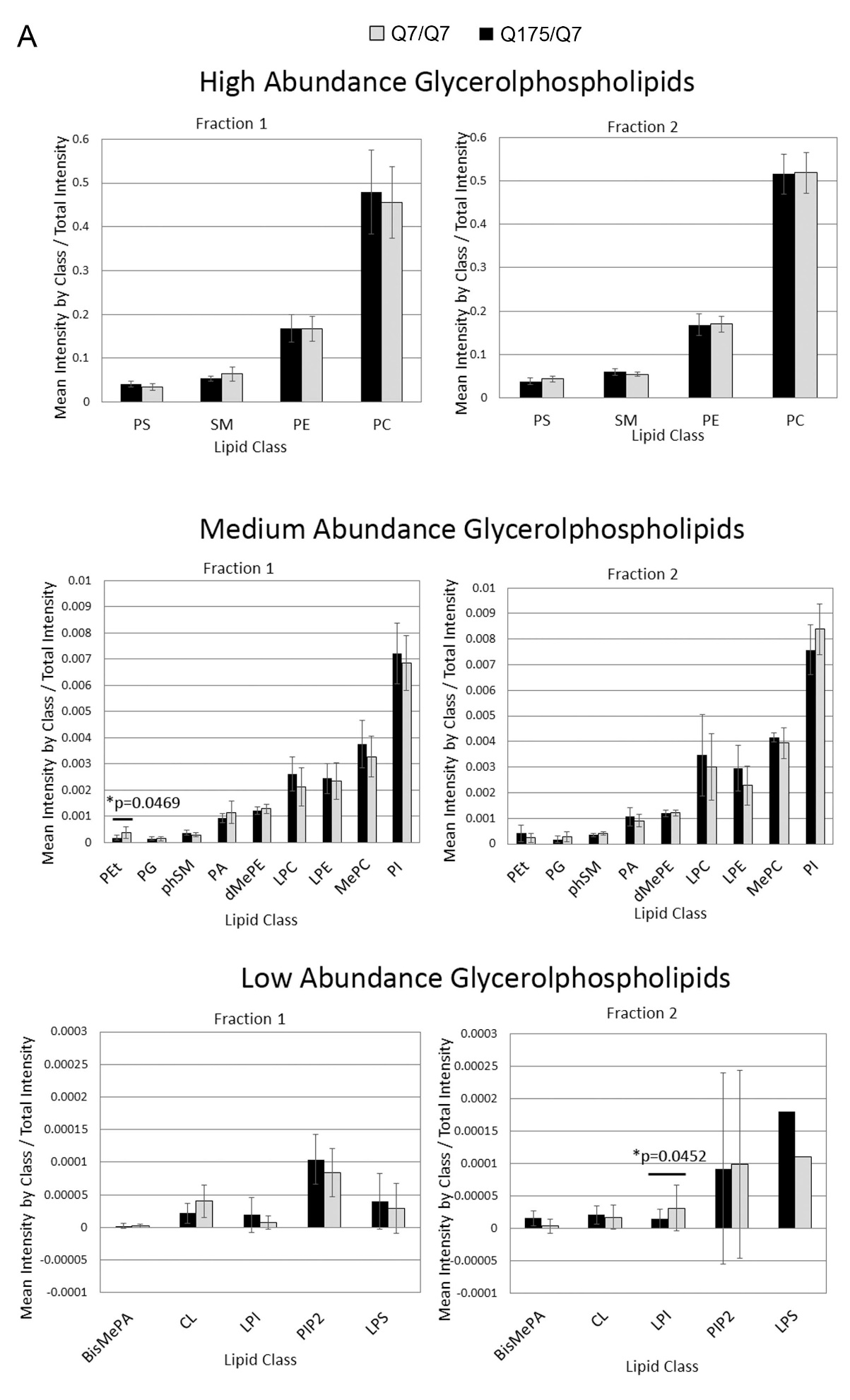


**
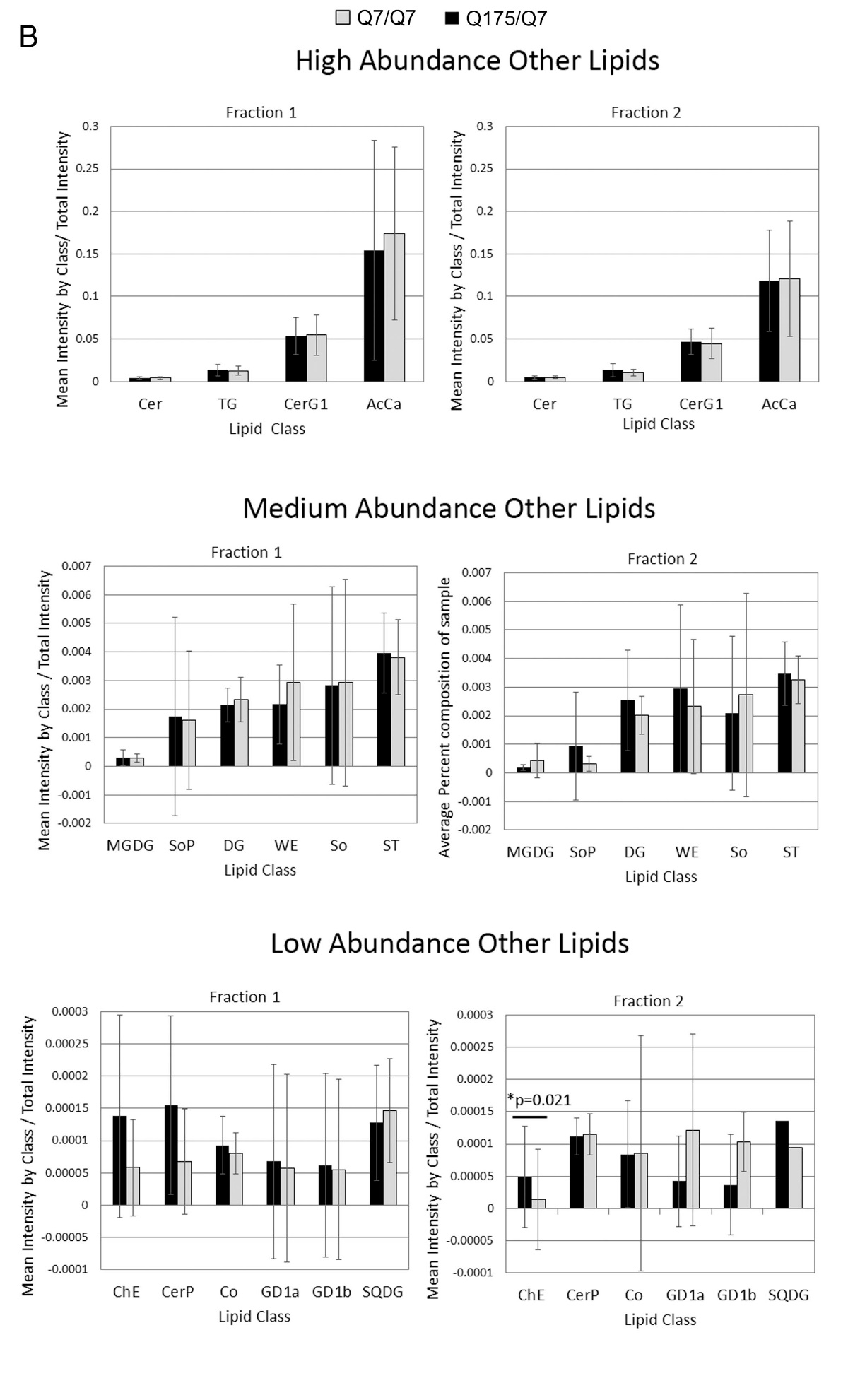
 Supplementary Figure 4. Levels of (A) glycerophospholipids by class and (B) other lipids detected by LC-MS/MS in fractions 1 and 2 of continuous density gradients of fractionated Q175/Q7 and Q7/Q7 striata at 6 months.** Lipid were isolated from 100 ul of fractions 1 and 2 using MBTE and analyzed by LC-MS/MS. Bar graphs ±SD show mean intensity for the sum of lipids within each class as a percent of total lipids detected. Lipids are grouped by their relative abundance and compared between WT (white bars) and HD mice (gray bars) (N=9 per genotype; unpaired t-test). When considered by subclass, levels of high abundant lipids in the fractions are not significantly different between WT and HD. Three lipids of low/medium abundance were significantly different between WT and HD (ChE, Cholesterol ester; Pet, phosphatidylethanol; LPI. lysophosphatidylinositol). See also Table 2.


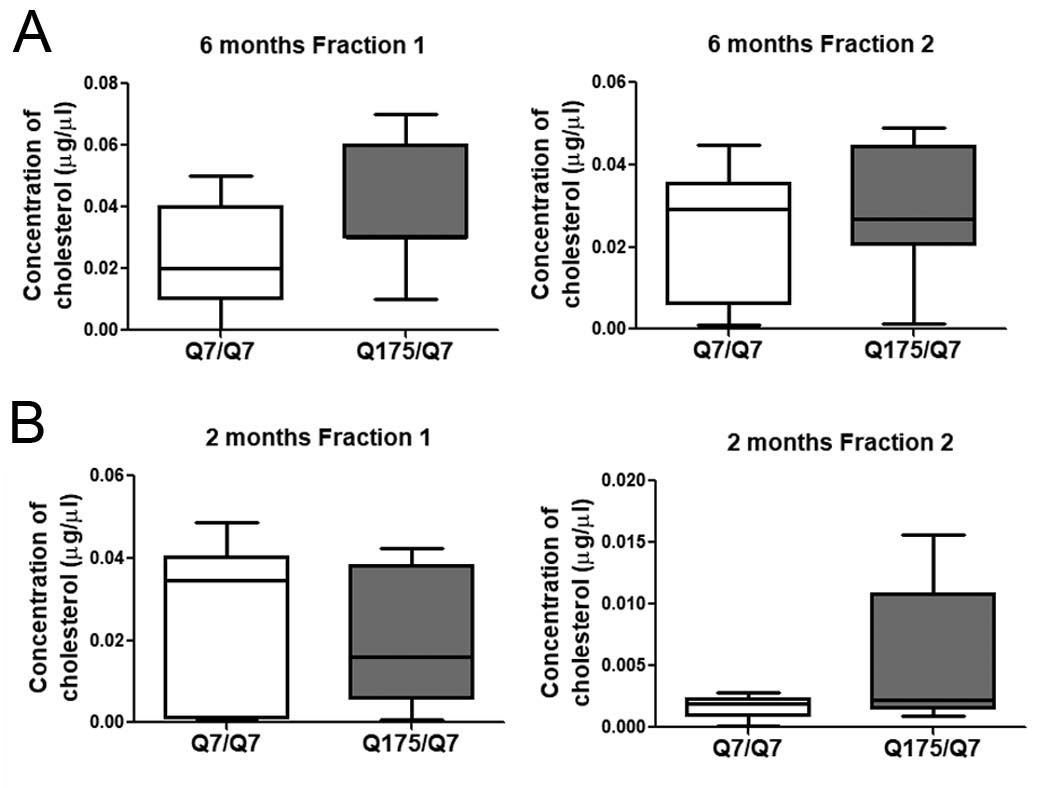


**Supplementary Figure 5. Levels of total cholesterol ester species measured using Amplex Red in fractions 1 and 2 of continuous density gradients of fractionated Q175/Q7 and Q7/Q7 striata.** After subcellular fractionation in a 0-35% continuous iodixanol gradient small amounts of fractions 1-2 were analyzed using the Amplex red cholesterol kit (3-5 μl for 6 month samples and 10 μl for 2 month samples). The Tukey style box plots show the lower and upper hinges corresponding to the first and third quartiles (the 25th and 75th percentiles) for both Q7/Q7 and Q175/Q7 in fractions 1 and 2 as described in Methods. There was no significant difference in cholesterol concentration between Q175/Q7 and Q7/Q7 in any of the fractions at 6 months (**A**, unpaired t-test, N=7) or 2 months (**B**, unpaired t-test, N=9). Note that the y-axis scales are different for each graph and for 6 months samples in A, insufficient material remained to perform this test for 2 pairs of mice.

**
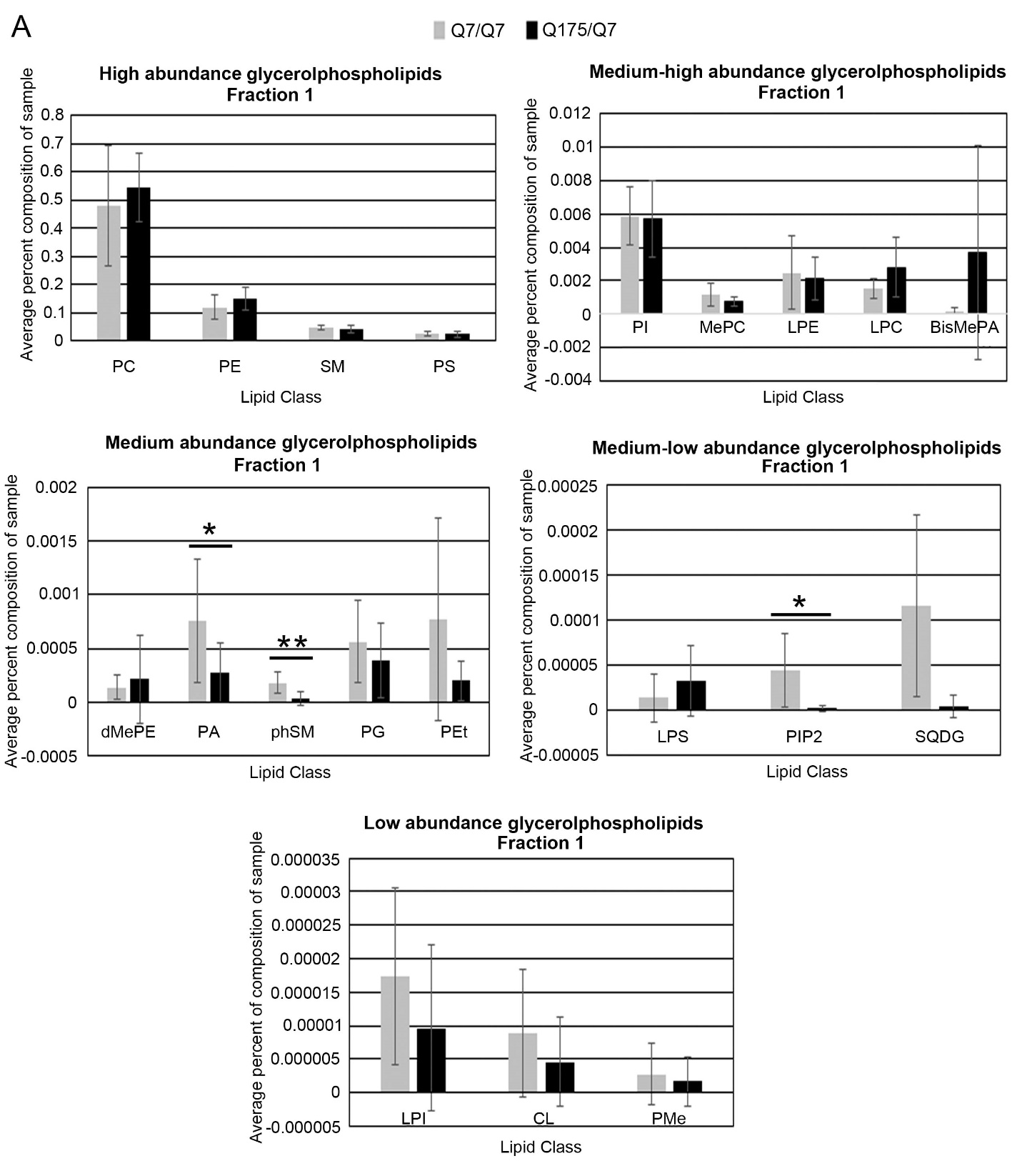
**


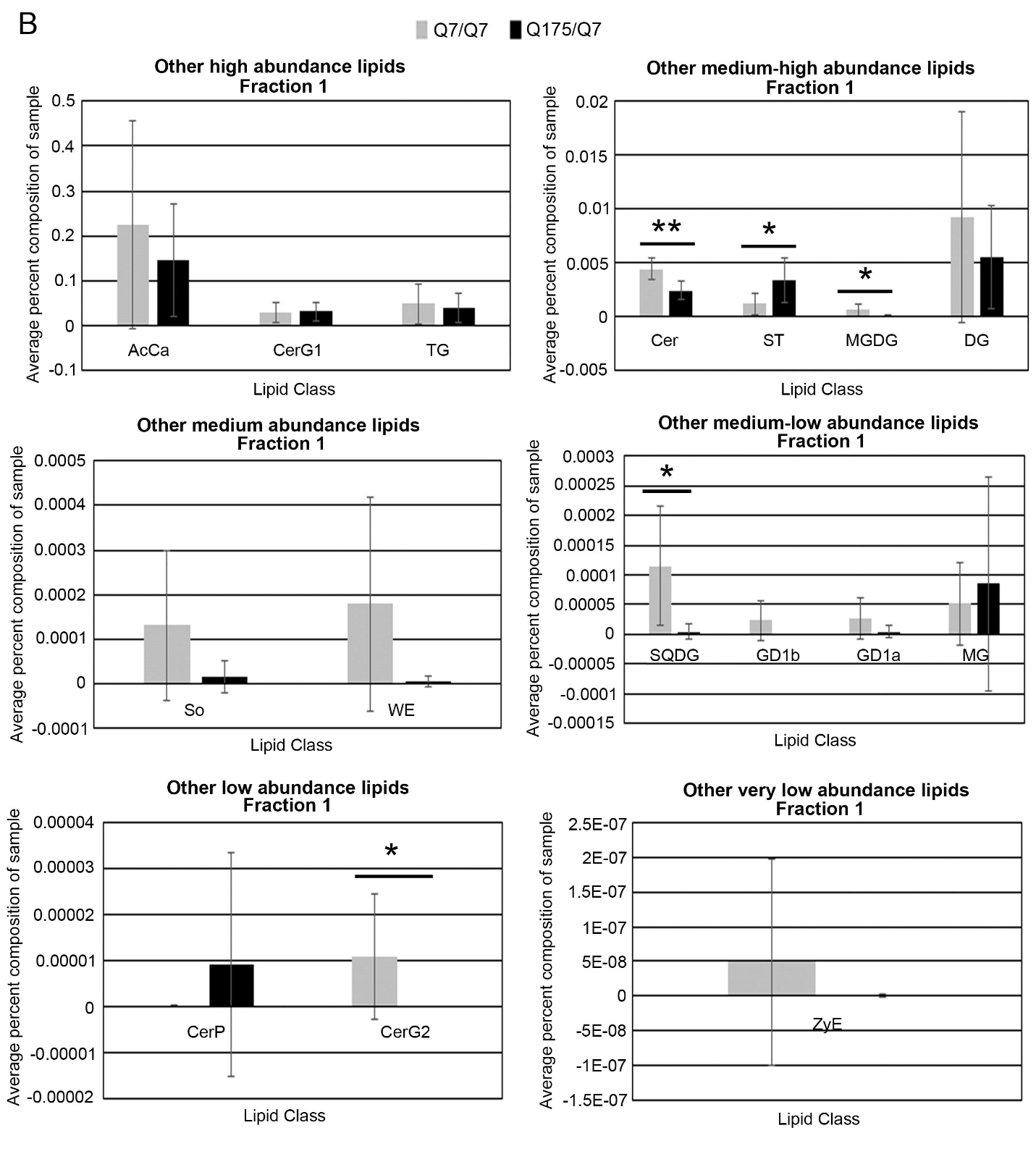


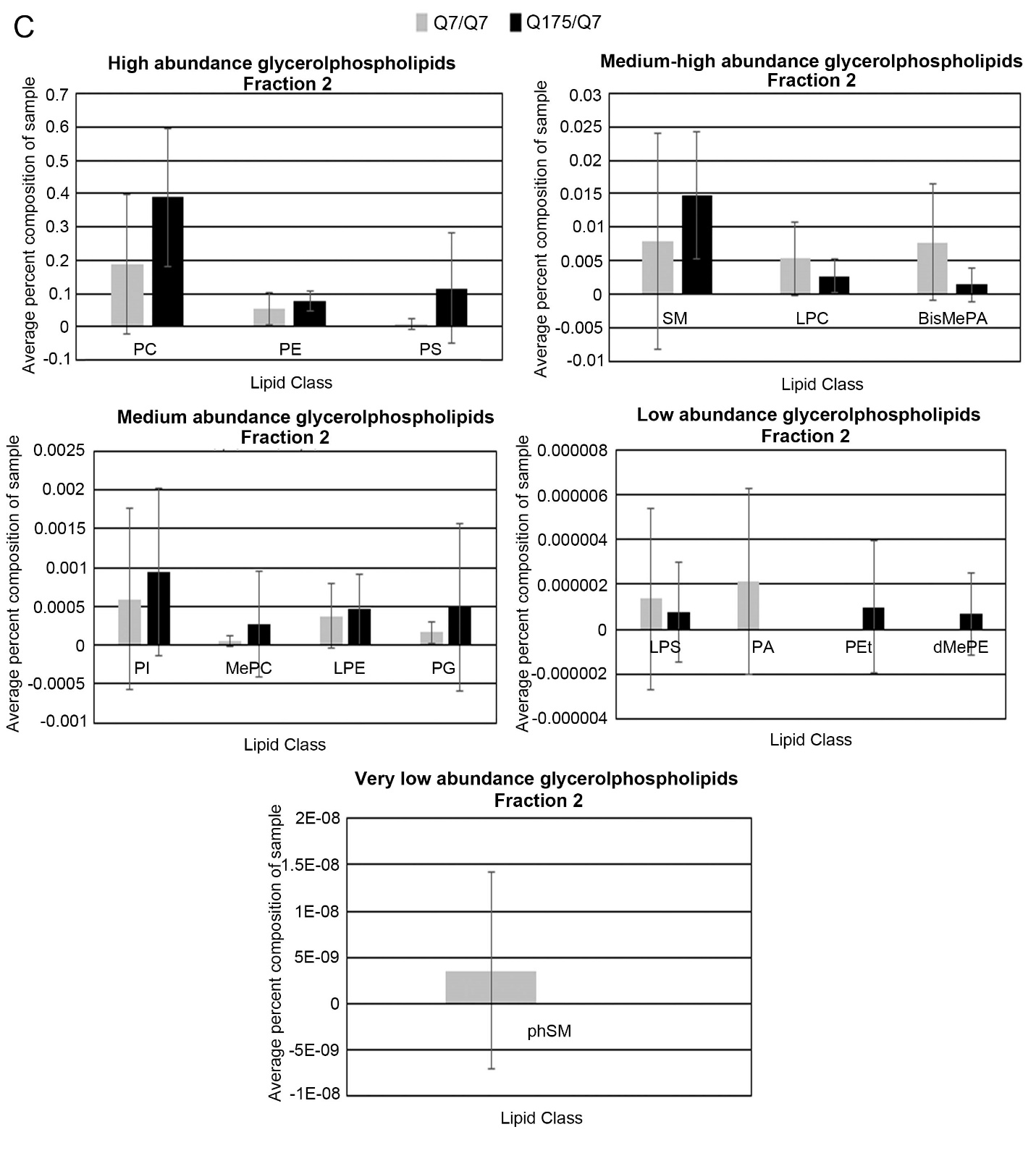


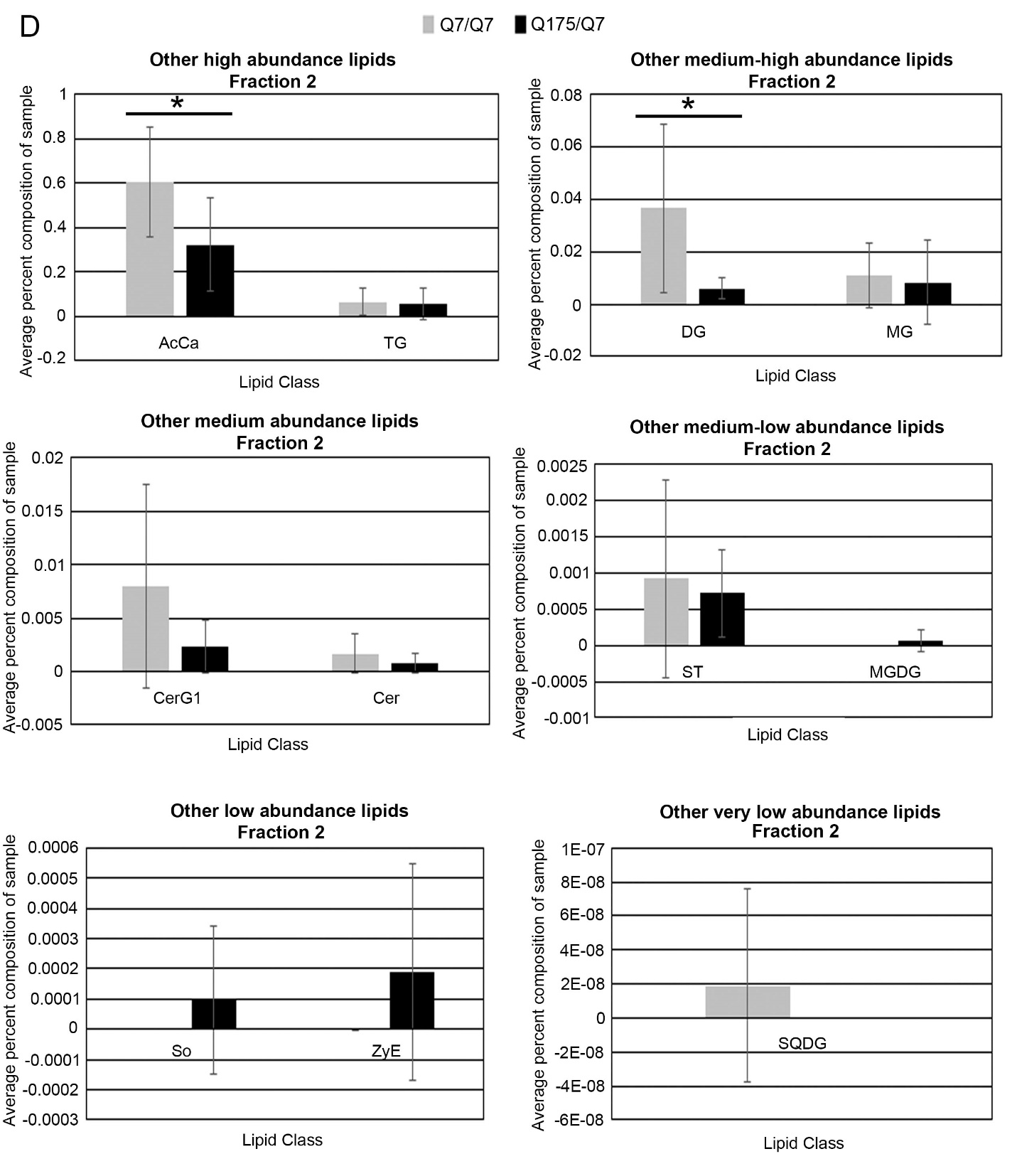


**Supplementary Figure 6. Levels of glycerophospholipids by subclass and other lipids detected by LC-MS/MS in fractions 1 and 2 of continuous density gradients of fractionated Q175/Q7 and Q7/Q7 striata at 2 months.** Lipid were isolated from 100 ul of fractions 1 and 2 using MBTE and analyzed by LC-MS/MS. Bar graphs ±SD show mean intensity for the sum of lipids within each class as a percent of total lipids detected. Lipids are grouped arbitrarily by their relative abundance and compared between WT (white bars) and HD mice (gray bars) (N=9 per genotype; unpaired t-test). **Supplementary Figure 4A.** Glycerophospholipids that were significant at 2 months in F1: PA (*p **=** 0.04229), phSM (**p = 0.001811), PIP2 (*p= 0.013661). **Supplementary Figure 4B.** Other lipids that were significant in F1at 2 months: Cer (**p= 0.000366), ST (*p = 0.016365), MGDG (*p =0.03814), SDDG (*p = 0.010991), CerG2 (*p = 0.044629). **Supplementary Figure 4C.** Glycerophospholipids that were significant at 2 months in F2**:** None. **Supplementary Fig 4D. Other lipids that were significant in F2:** Acca (*p = 0.02024), DG (*p = 0.020947). See also Table2.





**Supplementary Figure 7. Subcellular fractionation by density gradient ultracentrifugation of human putamen.**  Dorsal putamen was fractionated as described in Methods. Fractions 1-16 were removed from top to bottom. Fractions were assessed by SDS-PAGE and western blot analysis using the cell compartment markers indicated in Figure 6A to ensure complete separation. Equal volumes of each fraction were loaded per lane. Fractions 3-8 were analyzed in pairs and run on the same gel to eliminate variability due to different runs. Seven probes were analyzed by western blot for each gel (Calnexin, GLUT3, Na+/K+ ATPase, NMDAR 2b, PSD95, SNAP25 and XK). Band intensity for each fraction was standardized to sum of intensities for bands in fractions 3-8. Line graphs show mean percent of total signal ± SD obtained in fractions 3-8 for each fraction (N=3). There were no significant differences between control and HD for NaK ATPase, NMDAR 2b, SNAP25, or PSD95.
